# Dynamic Decoration of DNA Scaffolds for High‐Resolution Cancer Cell Subtyping

**DOI:** 10.1002/advs.202518307

**Published:** 2026-01-21

**Authors:** Xiaolin Hu, Jie Xie, Xinlin Guo, Liangting Wang, Zhengheng Yu, Xiaopei Qiu, Heng Li, Kang Wang, Xiaoxing Wang, Mingxuan Song, Junsong Guo, Wei Gu, Sergio Bernardini, Chaoyong Yang, Hong Zhang, Yang Luo

**Affiliations:** ^1^ Department of Laboratory Medicine Chongqing Center For Clinical Laboratory School of Medicine Chongqing Academy of Medical Sciences Chongqing General Hospital Chongqing University Chongqing P. R. China; ^2^ College of Bioengineering Key Laboratory For Biorheological Science and Technology of Ministry of Education Chongqing University Chongqing P. R. China; ^3^ Institute of Pathology and Southwest Cancer Center Southwest Hospital Third Military Medical University (Army Medical University) and Key Laboratory of Tumor Immunopathology Ministry of Education of China Chongqing P. R. China; ^4^ College of Life Science and Laboratory Medicine Kunming Medical University Kunming Yunnan P. R. China; ^5^ Department of Experimental Medicine University of Rome Tor Vergata Rome Italy; ^6^ The State Key Laboratory of Physical Chemistry of Solid Surfaces MOE Key Laboratory of Spectrochemical Analysis & Instrumentation Key Laboratory for Chemical Biology of Fujian Province Department of Chemical Biology College of Chemistry and Chemical Engineering Xiamen University Xiamen P. R. China

**Keywords:** cancer cell, DNA nanotechnology, imaging, self‐assembly, strand displacement

## Abstract

Precise imaging of cancer cells serves as the foundation for subtype analysis, significantly advancing the development of precision medicine. The unavoidable cellular internalization of fluorescent labels constrains the resolution and timeliness, presenting a significant obstacle. This study introduces a dynamic decorating strategy of DNA scaffolds that enables the execution of four distinct Boolean logic operations through self‐assembly and self‐disassembly. By integrating the in‐built molecular circuit, the proposed assay achieved signal‐amplified detection of low‐abundance nucleic acid inputs. In the application of cell imaging, inputs were labeled with aptamers to operate membrane‐confined self‐assembly or self‐disassembly of DNA scaffolds on the cell surface, enabling simultaneous identification of distinct subtypes of cancer cells with high fidelity. The intrinsic durability of DNA scaffolds successfully prevented cellular internalization for up to 300 min, boosting the long‐duration imaging. Moreover, the assay was capable of profiling a broad spectrum of cancer cell abundances from as low as 0.1% to 10% in clinical blood samples, consistently achieving recognition efficiency exceeding 60%. These findings underscore the transformational potential of DNA scaffold‐based imaging tools in biological research and precision medicine.

## Graphical abstract

This study presents a dual‐mode imaging platform that utilizes self‐assembly and disassembly of DNA scaffolds for high‐resolution cancer cell detection. The structural rigidity of DNA scaffolds effectively prevents cellular internalization for over 300 min. By integrating logic‐gated circuits, the system achieves specific profiling of cancer cell subtypes with a sensitivity down to 0.1%, offering a promising tool for precise diagnosis.

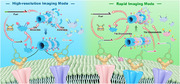

## 1 Introduction

Alterations in membrane protein expression significantly influence tumor behavior by regulating cancer cell growth and differentiation, leading to distinct cell subtypes with unique molecular characteristics and clinical outcomes [1, 2, 3]. Profiling membrane protein expression not only identifies specific cancer cell subtypes but also provides deep insights into the biological processes, progression mechanisms, and therapeutic responses of tumors [4, 5, 6]. Fluorescent antibody‐based methods, including immunofluorescence and flow cytometry, provide an efficient tool for membrane protein analysis and cancer cell identification [7]. However, these methods involve sequential and time‐consuming staining procedures and lack sufficient sensitivity to profile low‐abundance target cancer cells amidst a vast number of normal cells in complex physiological environments. Moreover, the inherent heterogeneity of cancer cells necessitates the simultaneous analysis of multiple membrane proteins to locate specific cancer cell subpopulations [8, 9] Therefore, developing a sensitive, and multiplexed membrane protein analysis technique for profiling cancer cell subtypes is crucial for tumor heterogeneity analysis, clinical diagnosis.

Aptamer‐based DNA molecular circuits, due to their high specificity and affinity, have been widely reported in cell subtyping, leveraging excellent programmability and optimal biocompatibility [10, 11, 12]. Among them, DNA logic circuits can perform computational operations at the molecular level to enable precise controls in response to specific stimulations through Boolean operations [13, 14, 15]. For instance, AND gate‐based strategies were used for sensitive analysis of multiple membrane receptors, employing aptamers as recognition tools and enzymatic [16, 17] or non‐enzymatic [18, 19, 20, 21, 22] circuits as signal amplifiers. However, DNA fluorescence probes used for live‐cell imaging are mostly small‐sized and less‐rigid [23, 24, 25]. These characteristics make them readily internalized by cancer cells through phagocytosis and caveolin‐mediated endocytosis, significantly impacting the imaging resolution [26, 27] Additionally, prolonged sample storage (over 3 h) also leads to excessive cellular endocytosis of the less rigid DNA fluorescence probes, further influencing the imaging duration [17]. Therefore, exploiting a robust DNA nanostructure to perform molecular operations on the live‐cell membrane is critical for high‐resolution and long‐duration membrane protein imaging and cancer cell subtyping.

DNA tile assembly, characterized by its formation of well‐defined structures through the organization of individual tiles represents a pivotal area of DNA nanotechnology, offering several distinct advantages in biomedical application [28, 29, 30]. First, the tile‐shaped nanostructure, affording numerous programmable sites, allows to precisely response various kinds of inputs via dynamic controls [31, 32] Second, DNA‐tile based scaffolds exhibit excellent rigidity to mitigate probe internalization compared with other DNA nanomaterials, such as proximity ligation clusters, ensuring the generation of low non‐specific signals in cell imaging [33, 34, 35, 36]. Additionally, the efficient assembly process contributes to a higher yield and a reduced complexity compared to other nanostructures, such as DNA origami. Recent studies have reported that DNA tile‐based scaffolds enable performing complex molecular operations for biomarker detection and drug delivery, yet their application in bioimaging remains limited [37, 38, 39]. Therefore, the main challenge is how to build a programmable nanoplatform suitable for cell membrane protein imaging combining the inherent advantages of DNA tile self‐assembly with aptamer‐based logic gates.

In this study, we introduced two dynamic and controllable decoration modes of DNA scaffolds, termed self‐driven assemble (SDA) and self‐driven disassemble (SDD), for rapid, sensitive and high‐fidelity responses to various kinds of inputs, such as nucleic acids and cancer cells. The de novo design leverages DNA tile assembly to ensure robust logic operation, and integrates DNA molecular circuits to enable sensitive and stable response to low‐abundance inputs. Moreover, we developed SDA‐based Dual‐Receptor Imaging (SDA‐DRI) and SDD‐based Triple‐Receptor Imaging (SDD‐TRI) systems for high‐resolution profiling of cancer cell subtypes by integrating the inputs with aptamers. Among these, SDA‐DRI approach could distinguish different abundance of leukemia cells ranging from 0.1% to 10% in blood samples within 100 min, and achieved differentiated imaging of four distinct leukemia cell subtypes in a cell mixture. SDD‐TRI system enabled precise subtyping of breast cancer cells within 20 min by enhancing local concentration through cholesterol‐mediated spatial confinement effects. Additionally, SDD‐TRI was applied to analyze the membrane protein expression profiles of different breast cancer subtypes, highlighting its potential in biological research and precision medicine. Specially, we also confirmed that the rigid structure of DNA scaffolds allows continuous imaging for up to 300 min without probe internalization by cancer cells, providing superior stability.

## 2 Results and Discussion

### 2.1 The Workflow of Cell Imaging via DNA Scaffolds

**FIGURE 1:**
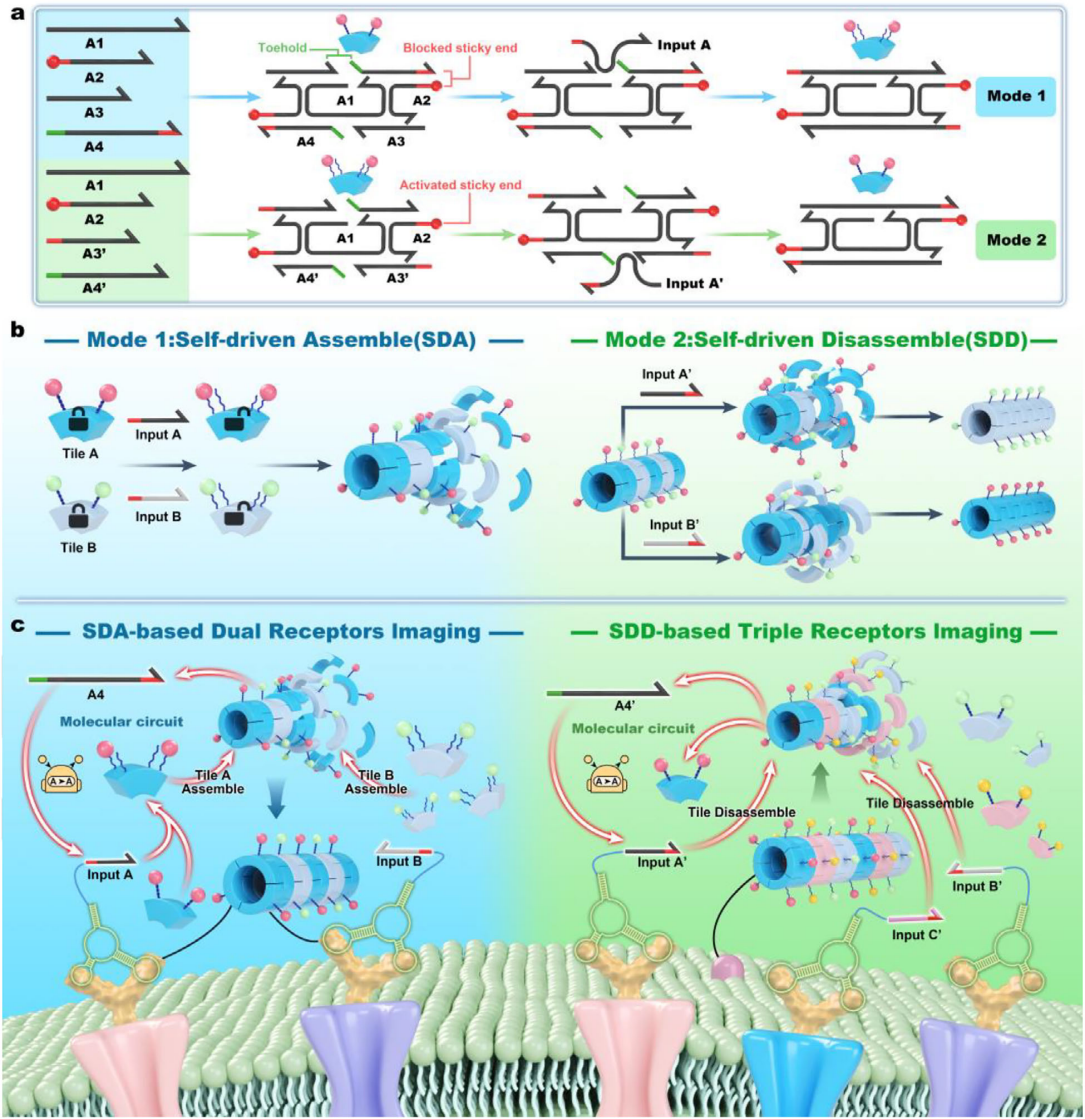
Design of the logic circuit and the mechanism of live cell imaging. (A) Assembly schematic diagram of DNA scaffolds and the structure of the two modes of DNA tiles. (B) The principle of input‐induced SDA and SDD. In the SDA mode, the sticky end was positioned at the 5’ end of the Input strand, which complemented the 3’ end and unlocked the 5’ end of the SDA‐Tile, thereby facilitating the self‐assembly of DNA scaffolds. In contrast, the sticky end is located at the 3’ end of the Input’ in the SDD mode, locking the SDD‐Tile and promoting the disassembly of DNA scaffolds. (C) Schematic illustrations for SDA‐DRI and SDD‐TRI systems.

DNA scaffolds, formed by the self‐assembly of DNA tiles, were served as the basic unit of logic circuits, exhibiting remarkable programmability [40]. We designed two modes to execute dynamic decoration of DNA scaffolds, termed Self‐Driven Assembly (SDA) and Self‐Driven Disassembly (SDD). The SDA‐Tiles, composed of A1, A2, A3, and A4, were initially in a blocked state. The input‐driven toehold‐mediated strand displacement (TMSD) triggered the unlocking of SDA‐Tiles, enabling the precise self‐assembly into a DNA scaffold. SDD‐Tiles, composed of A1, A2, A3', and A4', could be directly assembled into DNA scaffolds through annealing. The introduction of the input’ triggered TMSD to detach tiles from DNA scaffolds (Figure 1A). Further, we synthesized Cy5‐labeled DNA tiles (Tile A) and Cy3‐labeled DNA tiles (Tile B) to achieve multiplexed synchronous operations. Specially, these tiles had distinct framework sequences but shared the same sticky ends, enabling the execution of both SDA and SDD operations in response to different inputs (Figure 1B).

To address the low resolution caused by probe internalization in live‐cell imaging, we harnessed robust DNA scaffolds to develop two imaging modes with enhanced fidelity: SDA‐based Dual‐Receptor Imaging (SDA‐DRI) and SDD‐based Triple‐Receptor Imaging (SDD‐TRI), both supported by aptamer‐induced membrane receptor recognition. In the SDA‐DRI mode, two aptamers (labeled with input A and B) are designed to recognize distinct membrane proteins. These input strands trigger the corresponding TMSD, forming fluorophore‐labeled DNA scaffolds to enable precise phenotyping. Additionally, the byproducts A4 and B4 from TMSD can be recycled into inputs A and B through a molecular circuit for signal amplification. In the SDD‐TRI mode, cholesterol‐modified triple‐color DNA scaffolds (CTDs) are employed to anchor target cells. Distinct membrane proteins allow various input’ strands to be anchored on the cell surface and execute SDD of CTDs, thereby enabling the target cells to display distinct fluorescent labels in flow cytometry or confocal microscopy (Figure 1C).

### 2.2 Mechanism and Validation of SDA and SDA‐Based Logic Gates

Four distinct DNA tiles with different fluorescent labels and sticky end states were designed for the SDA (SDA‐Tile A and SDA‐Tile B) and SDD (SDD‐Tile A and SDD‐Tile B) modes (Figure S1) [41]. Optimization of the assembly time of DNA scaffolds revealed 30 h as optimal, with atomic force microscopy and transmission electron microscope confirming successful formation of tubular structures (Figures S2–S4). The stability analysis revealed that DNA tiles maintained slow degradation kinetics over 120 min, supported by inter‐strand stacking interactions. In comparison, the compact architecture of DNA scaffolds conferred stronger nuclease resistance, enabling stable persistence in 10% FBS (Figure S5). These findings collectively highlighted the superior structural robustness of the scaffolds under physiological conditions.

Moreover, we identified a 7‐nt toehold length for optimal TMSD efficiency (Figure S6). To improve the sensitivity and programmability, we designed four molecular circuits to translate A4 or B4 to input A or B as needed. The gel analysis validated the successful operation of the molecular amplifiers. Additionally, fluorescence kinetics analysis revealed that high concentrations of Mg^2+^ inhibited TMSD, probably due to competitive binding with ligands, while lower concentrations resulted in incomplete reactions [42]. Finally, 60 mM was determined for the subsequent steps (Figure S7).

**FIGURE 2:**
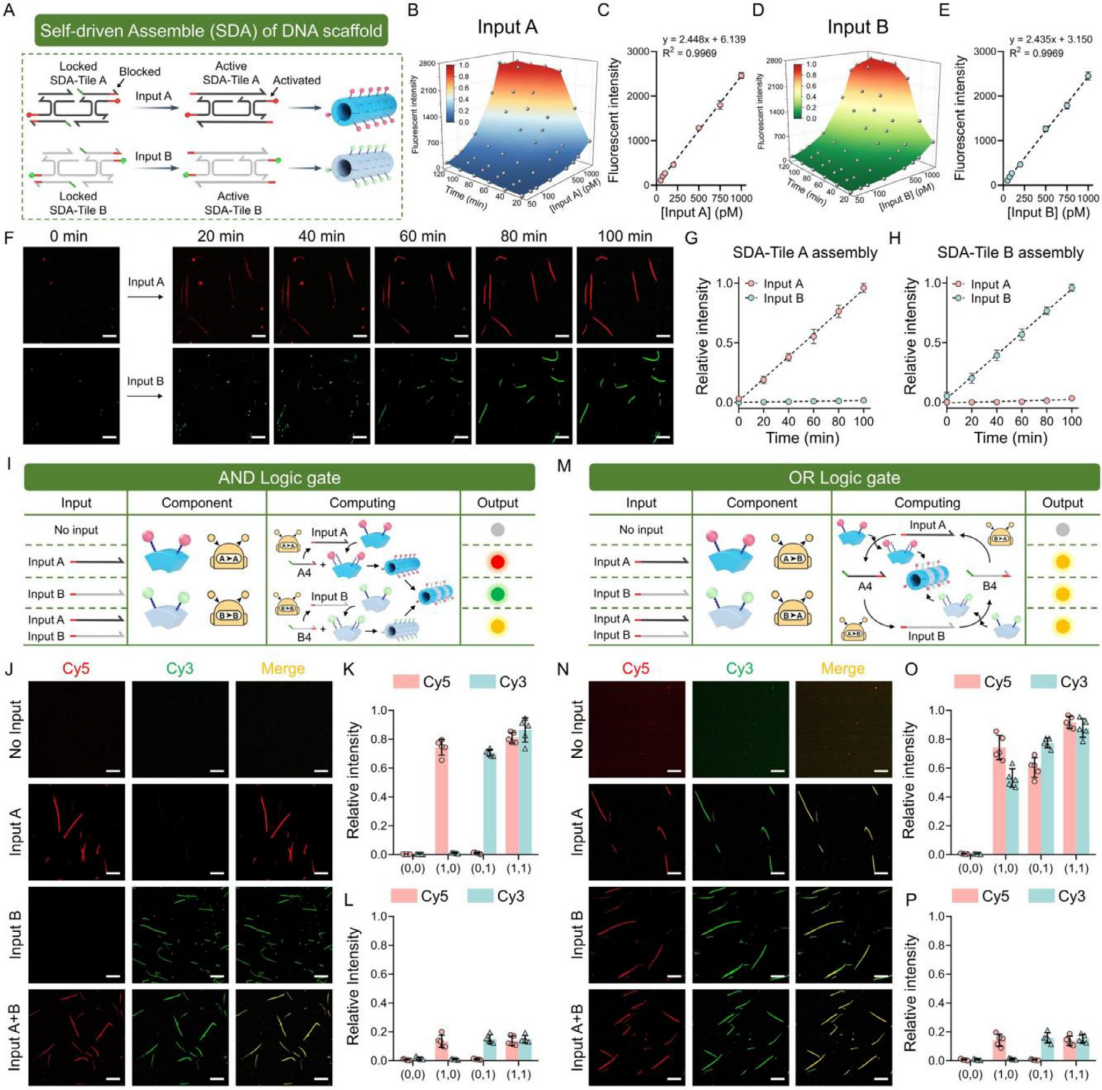
Construction of SDA‐based logic gates for the response to low‐abundance nucleic acid inputs. (A) Schematic diagram of SDA procedures. (B, D) Titration curves plotted at various time with different concentration of input A and B. (C, E) The sensitivity curves of inputs A and B at 100 min of reaction time. (F) Confocal images and (G, H) Relative intensities of the SDA‐Tiles A and B after adding inputs. Scale bar: 10 µm. (I) Principle of AND logic operation. (J) Confocal images and (K) relative intensities of output by AND logic gate. Scale bar: 10 µm. (L) Relative intensities of the output from the AND logic gate without the molecular circuit. (M) Principle of OR logic operation. (N) Confocal images and (O) relative intensities of output by OR logic gate. Scale bar: 10 µm. (P) Relative intensities of the output from the OR logic gate without the molecular circuit. Data presented as mean ± SD, n = 5.

In the SDA mode, input strands were used to unlock SDA‐Tiles, promoting the assembly of DNA scaffolds (Figure 2A). We first performed a systematic calibration of the analytical performance with synthetic nucleic acid inputs under optimized conditions. The real‐time fluorescence titration curves of input A and input B indicated that SDA exhibits strong dependence on both the reaction time and input concentration. However, SDA produced excessive output with high‐concentration input strands after 120 min, presumably due to the depletion of DNA tiles. Therefore, we plotted the concentration‐response curve of 100 min to ensure optimal sensitivity, achieving a detection limit of 27.7 picomolar (pM) of input A, and 36.1 pM of input B (Figure 2B–E). When the molecular circuit was omitted, the limit of detection decreased to 184.8 pM and 192.7 pM, respectively, demonstrating its significant signal amplification efficiency (Figure S8). Subsequently, we observed the dynamic assembly of DNA tiles using fluorescence microscopy. The result demonstrated that SDA‐Tiles appear as fluorescent spots in the initial state. After adding the corresponding input strands, the spots gradually aggregate to form tubular nanostructures with the reaction time increased from 0 to 100 min, while incorrect inputs failed to trigger the assembly of DNA scaffolds (Figure 2F–H). Furthermore, we successfully monitored the co‐assembly of the dual‐color DNA scaffold upon the simultaneous addition of input strands A and B in 100 min (Figure S9).

To demonstrate that DNA scaffolds could perform complex molecular computations through dynamic decorations, we constructed AND and OR logic gates based on SDA approach. The co‐assembly of SDA‐Tile A and SDA‐Tile B was defined as output 1, and the assembly of none or a single SDA‐Tile as output 0. In the AND gate, molecular circuits A to A and B to B were employed to ensure an output of 1 exclusively when both inputs A and B are present, as evidenced by observing dual‐color DNA scaffolds (Figure 2I). Fluorescence microscopy and statistical analysis of relative intensity demonstrated that the AND logic gate functioned properly, but fails to produce the correct truth value in the absence of molecular circuits (Figure 2J–L; Figure S10). Conversely, the OR gate utilized molecular circuits A to B and B to A to ensure either input could initiate the SDA reaction (Figure 2M). We successfully validated its capability to output the correct signal. (Figure 2N,O). Similarly, the OR gate failed to output the correct truth value in the absence of the molecular circuit (Figure 2P; Figure S11).

### 2.3 Mechanism and Validation of SDD and SDD‐Based Logic Gates

**FIGURE 3:**
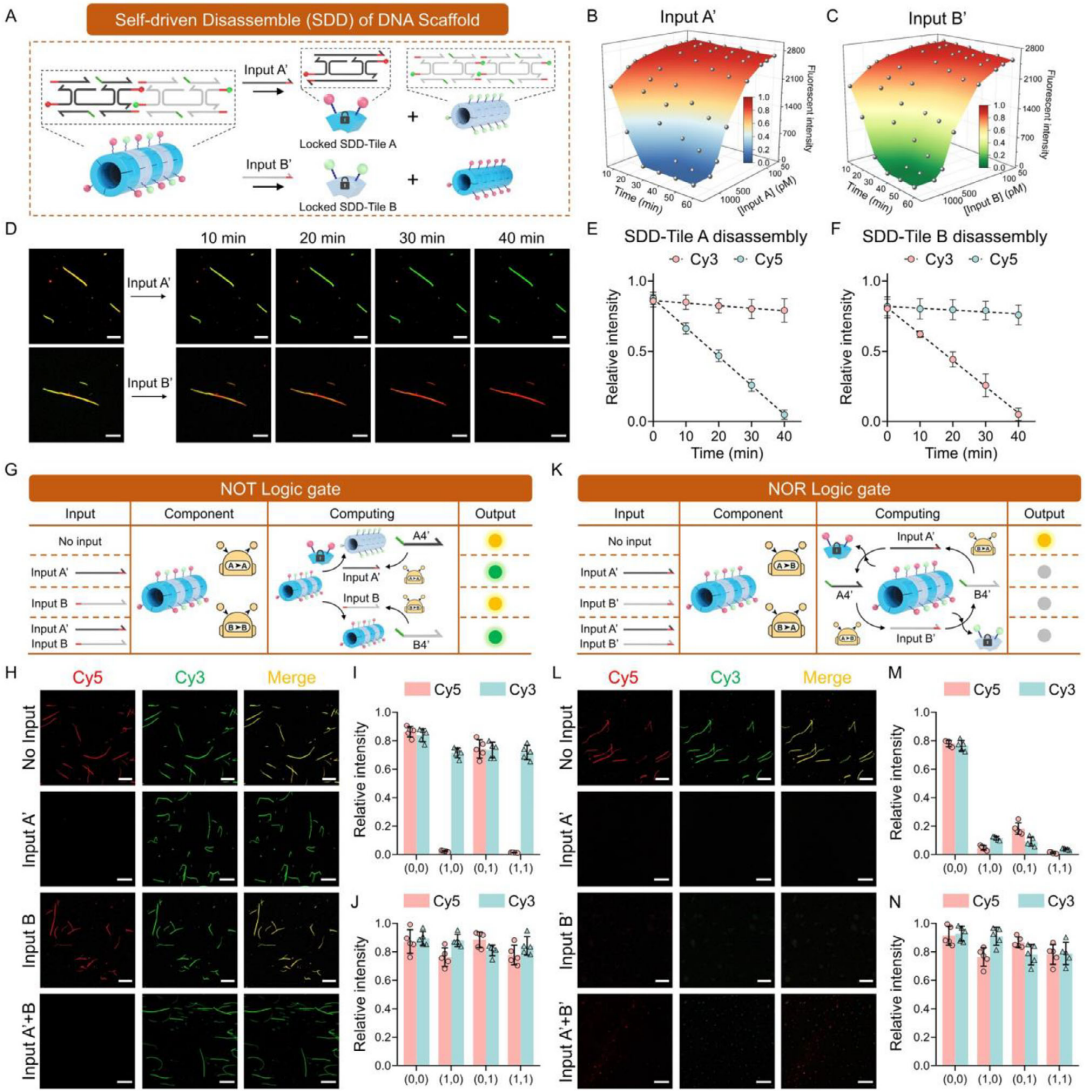
Construction of SDD‐based logic gates for the rapid response to nucleic acid inputs. (A) Schematic diagram of the SDD process. (B, C) Titration curves plotted at various time with different concentration of input A’ and B’. (D) Confocal images and (E, F) Relative intensity changes of the dual‐color DNA scaffolds after the addition of input A’ and B’. Scale bar: 10 µm. (G) Principle of NOT logic operation. (H) Confocal images and (I) relative intensities of output by NOT logic gate. Scale bar: 10 µm. (J) Relative intensities of the output from the NOT logic gate without the molecular circuit. (K) Principle of NOR logic operation. (L) Confocal images and (M) relative intensities of output by NOR logic gate. Scale bar: 10 µm. (N) Relative intensities of the output from the NOR logic gate without the molecular circuit. Data presented as mean ± SD, n = 5.

To validate the flexible design of DNA scaffold‐based logic circuits, we further presented the SDD mode, in which DNA tiles could form DNA scaffolds in the initial state and disassembled with the introduction of input’ (Figure 3A). We plotted concentration and time‐dependent titration curves for various input strands to systematically verify the performance. The results showed that higher concentrations and longer reaction times led to higher efficiency of SDD reactions (Figure 3B,C). The SDD mode can detect as low as 40.2 pM of input A and 45.6 pM of input B, as demonstrated by the titration curves at 40 min (Figure S12). We found that SDD exhibited lower sensitivity than SDA, which may attribute to the hidden toehold region within the structure of DNA scaffolds, thereby reducing the sensitivity of SDD in response to the input. However, the SDD system demonstrated a faster reaction process compared to SDA, as it required only a single TMSD step to complete all operations, whereas SDA involved an additional DNA tile self‐assembly step. Moreover, fluorescence microscopy and relative intensity statistics were employed to analyze the dynamic disassembly process of DNA scaffolds within 0 to 40 min. The results indicated that TMSD drove the detachment of Cy5 and Cy3‐labeled tiles from DNA scaffolds upon the addition of input A’ or input B’ respectively, whereas DNA scaffolds without the toehold domain failed to disassemble (Figure 3D–F; Figures S13, and S14). We then described NOT and NOR logic gates, where the initial output was 1 without any input. In the NOT gate. Input B’ was reprogrammed as a sequence without the blocking end, making it unable to disassemble SDD‐Tile B from DNA scaffolds, while input A’ remained unchanged (Figure 3G). In the NOT logic gate, molecular amplifiers for A to A and B to B are employed for signal amplifications. Fluorescence microscopy images unambiguously revealed the correct operation of the NOT logic gate and presented excellent responsiveness (Figure 3H,I). The input strand could only produce an undistinguishable color intensity change without molecular amplifiers, and the output was still 0 (Figure 3J; Figure S15). We also constructed a NOR logic gate by extending this concept to the OR logic gate, where any input could fully disassemble DNA scaffolds through molecular circuits (Figure 3K). Fluorescence microscopy images and relative intensities validated that the NOR gate operated correctly in the presence of molecular amplifiers, but fails to produce the correct output in their absence (Figure 3L–N; Figure S16).

### 2.4 Validation of DNA Scaffolds to Prevent Cellular Internalization

**FIGURE 4:**
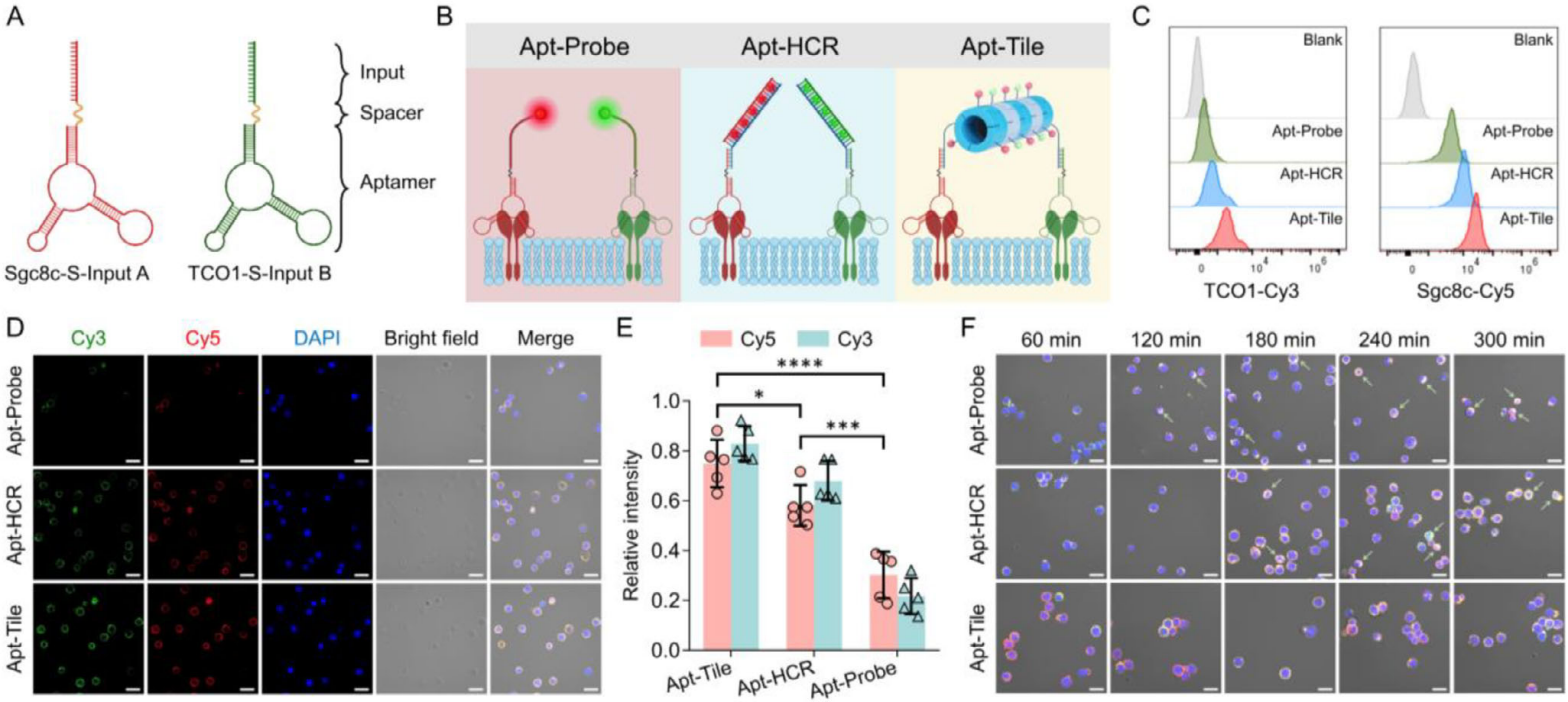
DNA scaffold‐based logic circuit for robust and long‐duration imaging of live cell. (A) Structure and functional domains of aptamer‐spacer‐input probe. (B) Scheme of Apt‐Probe, Apt‐HCR and Apt‐Tile for membrane protein recognition. (C) Flow cytometry analysis for the recognition of target cells with Apt‐Probe, Apt‐HCR, and Apt‐Tile. (D) Confocal images and (E) relative fluorescent intensities of three labelling methods for cell identifications. Scale bar: 20 µm. (F) Confocal images of target cells incubated for 60 to 300 min via Apt‐Probe, Apt‐HCR, and Apt‐Tile. Scale bar: 20 µm. Data presented as mean ± SD, n = 5. *p*‐values are calculated using one‐way ANOVA, ****p < 0.0001, ***p < 0.001, *p < 0.05.

The pronounced anti‐internalization of DNA scaffolds is attributed to their high‐aspect‐ratio, hollow geometry, which is unfavorable for endocytic uptake, combined with their exceptional structural rigidity that resists non‐specific cellular entry, ensuring minimal background in membrane‐based imaging [26, 34, 43] To validate this mechanism, we selected human acute lymphoblastic leukemia cells (CEM) as a model cell line and designed two aptamers targeting distinct membrane proteins: Sgc8c, which bound to protein tyrosine kinase 7 (PTK7), and TCO1, which recognized an unknown overexpressed marker [44, 45] Different aptamers were labeled with distinct inputs to activate SDA procedure, forming DNA scaffolds on the cell membrane (Figure 4A). The spacer length was optimized to minimize steric hindrance, ensuring the maximum self‐assembly efficiency. Notably, the highest signal intensity was observed with spacers longer than 15 nucleotides (Figure S17). Comparative analysis was conducted using three common membrane protein recognition techniques: fluorophore‐modified aptamer probe (Apt‐Probe), aptamer‐based hybrid chain reaction (Apt‐HCR), and aptamer‐based tile assembly (Apt‐Tile) (Figure 4B). Flow cytometry analysis indicated that Apt‐Tile output increased signal of 5.96‐fold and 2.44‐fold compared to Apt‐Probe and Apt‐HCR, respectively, demonstrating the superior labeling efficiency of the SDA system (Figure 4C). Furthermore, fluorescence microscopy imaging confirmed that Apt‐Tile exhibited higher resolution, and enhanced the target cell signal by 3.81‐fold and 1.29‐fold compared to Apt‐Probe and Apt‐HCR, respectively (Figure 4D,E).

Subsequently, we further assessed the anti‐internalization properties of DNA scaffolds. Consistent with our hypothesis, no internalization was observed for Apt‐Tile even after 300 min, with fluorescence signals remaining precisely co‐localized at the cell membrane. In contrast, Apt‐Probe and Apt‐HCR exhibited enhanced probe internalization at 120 min and 180 min, respectively, with significant fluorescent signals observed in both the target cell cytoplasm and nucleus (Figure 4F). These findings underscored the enhanced imaging resolution and robust stability of the proposed assay, attributable to the stable rigid structure of DNA scaffolds.

### 2.5 High‐Resolution Imaging of Dual‐Receptor on Cell Membrane Using SDA Circuits

**FIGURE 5:**
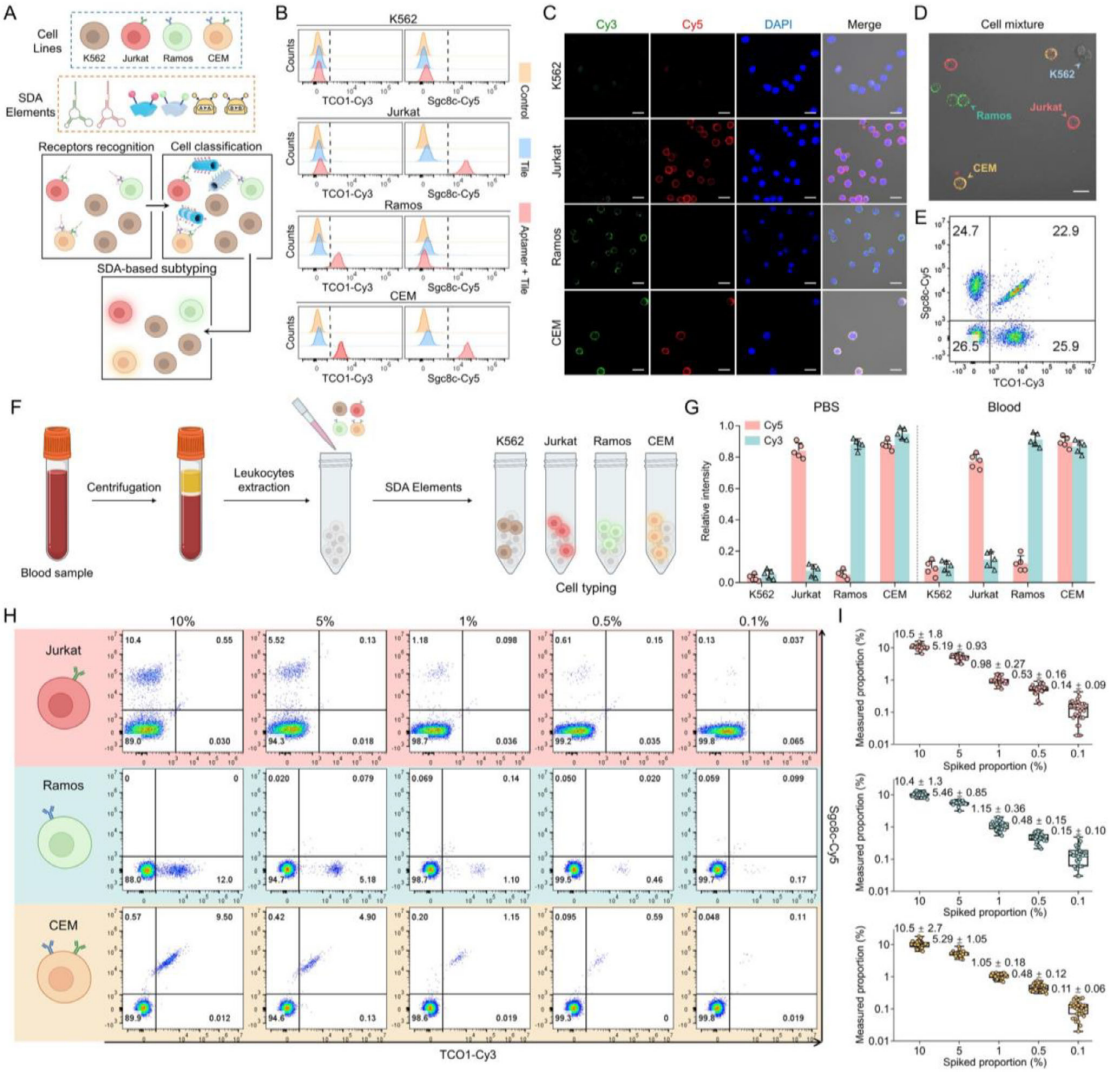
SDA‐DRI system for low‐abundance leukemia cell typing. (A) The standard SDA procedure for leukemia cell identification. (B) Flow cytometry analysis and (C) Confocal images of four leukemia cells treated with the SDA‐DRI components. Scale bar: 20 µm. (D) The classification of four leukemia cells in cell mixtures in the laser scanning confocal microscope. Scale bar: 20 µm. (E) Flow cytometry statistics of target cell signals in cell mixtures. (F) Experimental procedures of the SDA‐based subtyping of cancer cells in clinical blood samples. (G) The relative intensities of different cell lines in PBS and serum. Data presented as mean ± SD, n = 5. (H) Flow cytometry analysis of simulated clinical samples containing lymphocytes and 10 %, 5 %, 1 %, 0.5 %, and 0.1 % cancer cells. (I) Profiling different proportions of three leukemia cells spiked in blood samples before and after labelling with DNA scaffolds. Data presented as mean ± SD, n = 30.

To confirm that the SDA circuit functioned as a customizable and ultra‐sensitive molecular circuit for cancer cell subtyping, we constructed an SDA‐based dual‐receptor imaging (SDA‐DRI) assay tailored for high‐resolution leukemia cell imaging and subtyping. In addition to the dual‐positive cell line CEM, three control cell lines were included: human acute T‐cell leukemia cells (Jurkat) that bind exclusively to Sgc8c; human Burkitt lymphoma cells (Ramos) that bind solely to TCO1; and human erythroleukemia cells (K562) that do not bind to either [46]. The SDA‐DRI assay involved three steps: (1) aptamer‐modified inputs were added to target cells to enable membrane receptor recognition. (2) AND logic components were introduced for DNA tile assembly and signal amplification. (3) confocal microscopy was employed for cell typing (Figure 5A). Flow cytometry and fluorescence microscopy results demonstrated that SDA‐DRI assay effectively generated distinguishable fluorescent signals for distinct cancer cell subtypes, confirming its clinical potential for cell profiling (Figure 5B,C; Figure S18). Substitution of the input strands with random sequences resulted in an incorrect output to the target cells, validating its high specificity (Figure S19). To test the reliability of the SDA‐DRI model for accurately quantifying each target cell type in mixed samples, equal quantities of the four cell types were combined. The fluorescence microscopy images showed precise and simultaneous identification of the four distinct cell lines (Figure 5D). Flow cytometry further confirmed the proportional representation of each cell type within the complex environment (Figure 5E). Additionally, OR gate‐based SDA‐DRI of leukemia cells was constructed using molecular amplifiers of A to B and B to A. Flow cytometry and fluorescence microscopy results confirmed that cells containing any of the membrane proteins exhibited fluorescence co‐localization, while maintaining high imaging resolution (Figure S20).

In the early stages of cancer, cancer cells are present in minuscule quantities and the internal environment is more intricate. Therefore, we collected 90 blood samples from healthy individuals to verify whether SDA‐DRI assay could track cancer cells in such complex settings. First, blood samples were centrifuged to obtain lymphocyte populations. After that, various leukemia cells were introduced to simulate cancerous conditions. Lastly, tile components were added to the simulated clinical samples and carried out high‐resolution identification through SDA‐DRI assay (Figure 5F). The result demonstrated that SDA‐DRI system enable to discriminate distinct tumor cell subtypes (Figure 5G).

Subsequently, we introduced various proportions (10%, 5%, 1%, 0.5%, 0.1%) of Jurkat, Ramos, and CEM into the blood samples to assess the sensitivity. Flow cytometry analysis revealed that the SDA‐DRI strategy successfully discriminated Jurkat, Ramos, and CEM cells across a broad concentration spectrum, maintaining accurate identification even at abundances as low as 0.1% (one cancer cell per one thousand normal cells) (Figure 5H). Given that the tumor burden at diagnosis typically far exceeds this level, the achieved sensitivity of 0.1% is clinically adequate for reliable cancer cell detection and aligns with the conventional flow cytometry. To ensure the robustness of the results, each proportion was tested in 30 independent replicates. The results indicated that the proposed strategy exhibited high reliability, consistently achieving leukemia cell recognition efficiencies exceeding 60% (Figure 5I; Figure S21).

### 2.6 SDD‐Based Triple Receptors Imaging for Rapid and Sensitive Breast Cancer Cell Subtyping

**FIGURE 6:**
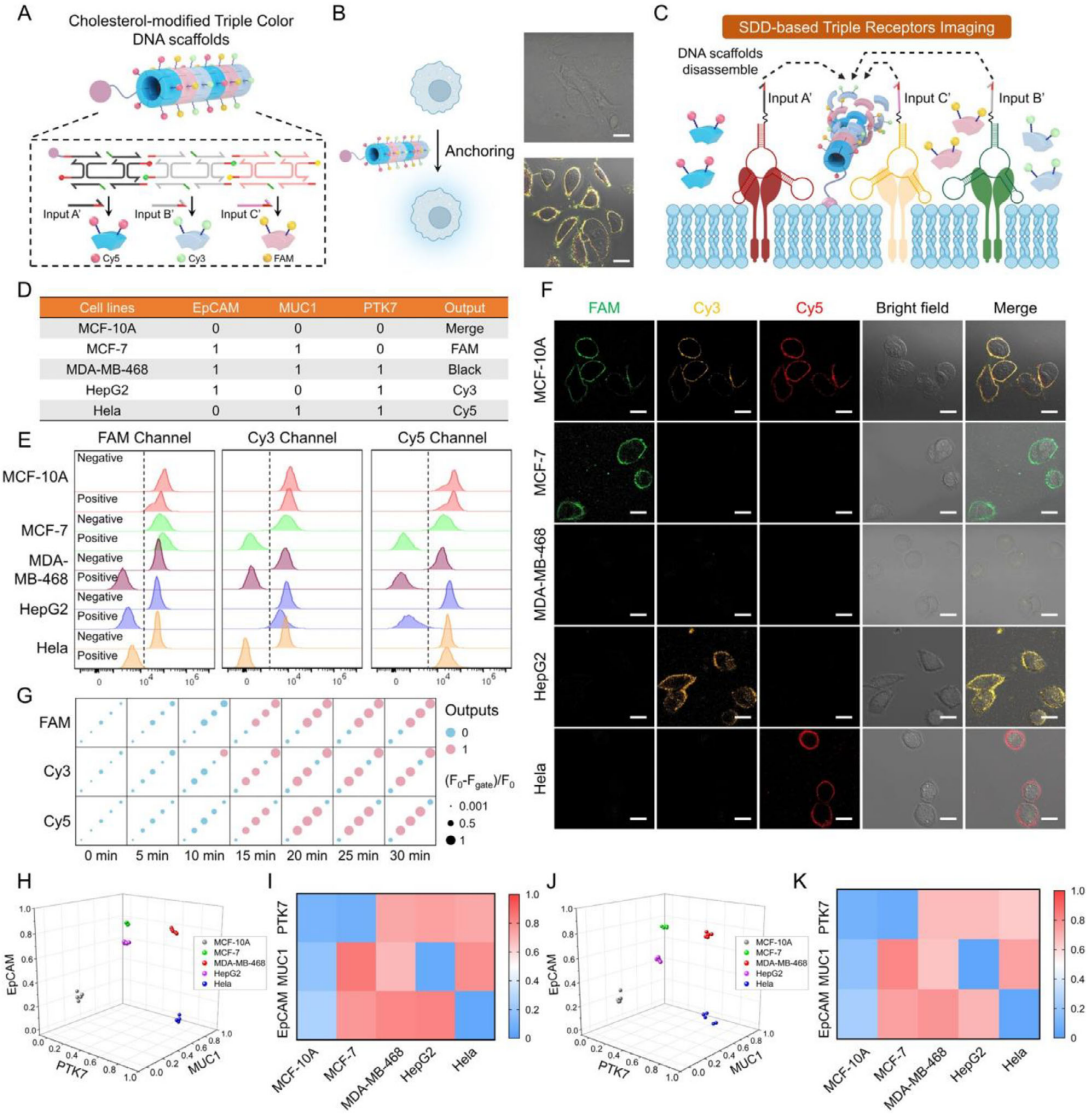
The design of SDD‐TRI for breast cancer cell typing. (A) Structure of CTDs. (B) Confocal analysis of the anchoring of CTDs to cancer cells. Scale bar: 10 µm. (C) Scheme of the triple receptor logic device for breast cancer cell typing. (D) Membrane protein expression profiles and theoretical output values of five different cell lines, including human breast epithelial cells (MCF‐10A), human breast cancer cells (MCF‐7), triple‐negative breast cancer cells (MDA‐MB‐468), human liver cancer cells (HepG2) and human cervical cancer cells (HeLa). (E) Flow cytometry analysis and (F) confocal images of five breast cancer cell lines with input‐mediated the disassembly of CTDs. Scale bar: 10 µm. (G) Flow cytometry‐based heatmap analysis of relative fluorescence intensity of five cell lines. (H, I) The 3D scatter plot and heat map of expression levels of PTK7, EpCAM, and MUC1 proteins on different kinds of cancer cells in PBS and (J, K) in serum.

Cell membranes exhibit fluid properties that enhance molecular collision efficiency within confined spaces [17]. Capitalizing on this, we designed CTDs to anchor cancer cells, where three types of DNA tiles tagged with distinct fluorescent groups and equipped with toehold domains (Figure 6A). The CTDs were first verified to respond precisely to various input combinations, producing distinct fluorescent readouts (Figure S22). The confined CTDs demonstrated high stability, resulting in high fluorescence signals at the membrane and minimal noise signals within the cells (Figure 6B). Flow cytometry analysis revealed that CTDs yielded over 20‐fold higher signal intensities compared to CTDs without cholesterol modification (Figure S23). Furthermore, we developed a rapid and high‐fidelity imaging mode called SDD‐based triple‐receptor imaging (SDD‐TRI). The SDD reaction was triggered by the aptamer‐modified input strands when receptors were present on the cell membrane, leading to the disassembly of corresponding fluorescent DNA tiles from CTDs. This mechanism allowed accurate cancer cell typing through simultaneous identification of multiple markers (Figure 6C).

As a proof‐of‐concept, SDD‐TRI was employed to profile breast cancer cells based on the characteristic proteins including EpCAM, MUC1, and PTK7 [10]. Cytotoxicity analysis first revealed excellent biosafety profiles, with cell viability of MCF‐10A maintaining above 90% after 12, 24, and 36‐hours exposure (Figure S24). Five cell lines (Breast cancer cells: MCF‐10A, MCF‐7 and MDA‐MB‐468, “bystander” cells: HepG2 and Hela) with different membrane protein profiles were included, and cell typing could be achieved by analyzing the fluorescence types on the cell surface (Figure 6D). The flow cytometry results revealed that all cell lines correspond to the setup described in Figure 6D. No background signals were observed in the negative controls, confirming the significant specificity of the SDD‐TRI (Figure 6E). Confocal images and fluorescent intensity analysis demonstrated that SDD‐TRI operated on cell membranes with high fidelity and effectively distinguished between different cell types (Figure 6F; Figure S25). Control experiments confirmed that scaffold disassembly was abolished when the input strands were replaced with random sequences (Figure S26). Additionally, we integrated the NOR gate components into the SDD‐TRI system to validate its potential for cancer cell screening. Flow cytometry analysis and confocal microscopy revealed that the NOR gate‐based SDD‐TRI effectively discriminates between normal cells and cancer cell lines with high accuracy (Figure S27).

We previously mentioned that SDD offered faster imaging times compared to SDA due to its more streamlined reaction process. To validate whether the viewpoint was suitable for cancer cell subtype identification, flow cytometry was employed to analyze the fluorescence signal changes across FAM, Cy3 and Cy5 channels for five different cell types over 0 to 30 min, allowing for a systematic examination of the reaction kinetics. Within 0 to 20 min, a significant decrease in fluorescence signals was observed in cell lines with the corresponding surface receptors, while those lacking the receptors remained unaffected. After 20 min, the rate of signal decline diminished, indicating that cell phenotype identification could be completed within 20 min (Figure 6G).

To explore the potential clinical applications of SDD‐TRI, the relative expression levels of membrane proteins were quantified in five cell lines. We first utilized a 3D scatter plot to illustrate the expression levels of membrane proteins, and the results revealed significant differences among the five cell lines (Figure 6H). The heatmap further confirmed that the protein expression levels were consistent with initial expectations (Figure 6I). Similar results were obtained in FBS, demonstrating the robustness of SDD‐TRI against interference (Figure 6J,K).

## 3 Conclusions

In summary, we introduced a DNA logic circuit utilizing DNA tile‐derived scaffolds, which offered both robustness and programmability, enabling precise imaging and subtyping of low‐abundance cancer cells. Two logic operational modes were designed: Self‐Driven Assembly (SDA), which guarantees enhanced fidelity, and Self‐Driven Disassembly (SDD), which facilitates expedited response times, allowing for tailored application according to unique requirements. Both SDA and SDD can respond to nucleic acid inputs at the picomolar level with the assistance of molecular amplifiers. In cell imaging, the stability of DNA scaffolds prevents internalization by cancer cells for up to 300 min, greatly improving imaging resolution. Furthermore, we constructed SDA‐DRI and SDD‐TRI systems for precise subtyping of lymphoma and breast cancer cells, respectively. The SDA‐DRI system demonstrated remarkable sensitivity, accurately identifying target cells at a 0.1% abundance and enabling the differentiation of four lymphoma subtypes in cell mixtures. The SDD‐DRI system utilized local confinement effects to enhance reaction concentrations and accelerate kinetics, enabling high‐resolution imaging and precise subtyping of five different cancer cell types within 20 min. The modular architecture of this platform, featuring swappable aptamer recognizers, toehold‐mediated triggers, and stable DNA tile scaffolds, ensures high generalizability for profiling diverse membrane biomarkers. Furthermore, the scalable logic framework can be readily expanded to accommodate additional inputs, paving the way for even more complex cellular diagnostic applications.

While the DNA scaffold‐based high‐resolution imaging strategy provides a promising platform for cancer diagnostics, several key challenges remain for the clinical translation. First, potential confounding factors present in actual patient samples have not yet been validated. Second, the standardization and scalable production of the DNA components for consistent clinical‐grade use requires further validation. Overcoming these hurdles is essential to transform this programmable nanotechnology into a reliable tool for pathological molecular subtyping, with potential future applications extending to liquid biopsy and intraoperative guidance.

## 4 Experimental Section

### 4.1 Synthesis of DNA Tiles

Taking the synthesis of SDA‐Tile A as an example, A1 (2 µM), Cy5‐labeled A2, along with A3 and A4 (4 µM), were mixed in 1 × TAE/Mg^2+^ buffer (40 mM Tris base, 20 mM acetic acid, 2 mM EDTA, and 12.5 mM magnesium acetate, pH 8.0, 50 µL). The solution was then heated to 95°C using a Mastercycler Gradient Thermal Cycler (Bio‐Rad, USA). After heating, the reaction mixture was annealed by cooling at a constant rate to 20°C, with annealing times set at 30 h, respectively. Following annealing, the SDA‐Tile A was purified using the HiPure DNA Clean Up Kit following the manufacturer's standard protocol, and stored at a concentration of 1 µM in Elution buffer at −20°C.

### 4.2 Construction of SDA‐Based Logic Gates

For the AND gate, SDA‐Tile A (200 nM), SDA‐Tile B (200 nM), molecular amplifier A to A and B to B (500 nM) were pre‐mixed in 1 × TAE/Mg^2+^ buffer (50 µL). For sensitivity, different concentrations of input A or input B (ranging from 50 pM to 2 nM) were added. For the AND gate, the input concentrations were varied as follows: 0 nM input A and B (No input group), 1 nM input A and 0 nM input B (Input A group), 0 nM input A and 1 nM input B (Input B group), and 1 nM input A and B (Input A + B group). Reactions were performed at 25°C for 100 min, after which images were captured using laser confocal microscopy. For the OR gate, the molecular amplifiers were replaced with A to B and B to A. The other steps remained the same.

### 4.3 Construction of SDD‐Based Logic Gates

A1 and B1 (2 µM), A2, A3’, A4’, B2, B3’, and B4’ (4 µM) were mixed in 1 × TAE/Mg^2+^ buffer (50 µL) to prepare dual‐color DNA scaffolds. For sensitivity, Dual‐color DNA scaffolds (200 nM), molecular amplifiers A to B and B to A (500 nM) and various concentrations of input A and B (ranging from 50 pM to 2 nM) were mixed. The reaction was carried out at 25°C for 0 to 60 min. For the NOT gate, input concentrations were varied as follows: 0 nM input A’ and B (No input group), 1 nM input A’ and 0 nM input B (Input A group), 0 nM input A’ and 1 nM input B (Input B group), and 1 nM input A'and B (Input A + B group). The reactions were carried out at 25°C for 40 min. For the NOR gate, input B was replaced with input B’, and molecular amplifiers were replaced with A to B and B to A in the above system.

### 4.4 Comparison of Different Imaging Methods

For Apt‐probe methods, CEM cell lines (1 × 10^5^) were washed twice with washing solution (4.5 g/L glucose, 5 mM MgCl_2_ in Dulbecco's phosphate‐buffered saline, 1 mL). A mixture of Cy5‐labeled Sgc8c‐probe and Cy3‐labeled TCO1‐probe (200 nM) was incubated with CEM cells in binding buffer (0.1 mg/mL yeast tRNA, 1 mg/mL BSA in washing buffer, 300 µL) at 4°C for 30 min.

For Apt‐HCR, Sgc8c‐S‐trigger and TCO1‐S‐trigger (200 nM) were incubated with CEM cells in binding buffer (300 µL) for 30 min. The cells were then washed three times, and incubated with Sgc8c‐H1, Sgc8c‐H2, TCO1‐H1, and TCO1‐H2 (500 nM) at 4°C for 60 min.

For Apt‐tile, cells labeled with Sgc8c‐S‐Input A and TCO1‐S‐Input B were incubated with SDA‐Tile A, SDA‐Tile B (500 nM), and molecular amplifiers A to A, and B to B (750 nM) in binding buffer (300 µL) at 4°C for 100 min.

### 4.5 SDA‐DRI Imaging System

Sgc8c‐S‐Input A and TCO1‐S‐Input B (300 nM) were incubated with K562 or Jurkat, Ramos, CEM cells (1 × 10^5^) in binding buffer (300 µL) at 4°C for 30 min. Subsequently, the cell suspension was incubated at 4°C for 100 min after adding SDA‐Tile A, SDA‐Tile B (500 nM), and molecular amplifiers A to A and B to B (750 nM). To validate the cell mixture, K562, Jurkat, Ramos, and CEM cells (2.5 × 10^4^) were mixed and processed as described above.

### 4.6 Clinical Sample Validation

For clinical sample simulation, 90 clinical blood samples were collected from Chongqing General Hospital with ethically obtained consent. all samples were from healthy individuals. The study was authorized by the Ethics Committee of Chongqing General Hospital and carried out in accordance with the 1964 Declaration of Helsinki and its subsequent amendments (ethical code KYS2025‐047‐01). All 90 blood samples were incubated with red blood cell lysis buffer at room temperature for 10 min, followed by centrifugation at 2500 rpm for 5 min. The precipitated white blood cells were harvested, washed three times with washing buffer, and then resuspended in the same buffer (200 µL). Then, each sample of white blood cells were separately spiked with Jurkat, Ramos, or CEM cells at proportions of 0.1%, 0.5%, 1%, 5%, and 10%, respectively. After the treatment with SDA components, all samples were subjected to flow cytometric analysis. The resulting dot plot was divided into four quadrants: Q1 (Sgc8c^+^/TCO1^−^), Q2 (Sgc8c^+^/TCO1^+^), Q3 (Sgc8c^−^/TCO1^+^), and Q4 (Sgc8c^−^/TCO1^−^). The recognition efficiency for each cell line was then calculated as the proportion of cells located in its diagnostic quadrant, normalized to the total population excluding double‐negative (Q4) events. The formulas used were:

(1) $$\mathrm{Recognition}\;\;\mathrm{efficiency}\;\;of\;\mathrm{Jurkat}\;=\frac{P_{Q1}}{1-P_{Q4}}$$

(2) $$\mathrm{Recognition}\;\mathrm{efficiency}\;\mathrm{of}\;\mathrm{Ramos}\;=\frac{P_{Q3}}{1-P_{Q4}}$$

(3) $$\mathrm{Recognition}\;\mathrm{efficiency}\;\mathrm{of}\;\mathrm{CEM}\;=\frac{P_{Q2}}{1-P_{Q4}}$$

where P represents the proportion of cancer cell in the indicated quadrants.

### 4.7 SDD‐TRI Imaging System

Cy5 and cholesterol‐labeled SDD‐Tile A, Cy3‐labeled SDD‐Tile B, and FAM‐labeled SDD‐Tile were mixed to prepare cholesterol‐labeled three‐color DNA scaffolds (CTDs). Next, EpCAM‐S‐Input A’, MUC1‐S‐Input B’, and PTK7‐S‐Input C’ (300 nM) were incubated with MCF‐10A or MCF‐7, MDA‐MB‐468, HepG2, HeLa cells (1 × 10^5^), followed by three washes to remove unbound aptamers. Subsequently, CTDs (500 nM) were added and incubated with the aptamer‐labeled cells at room temperature for 20 min. Various molecular amplifiers (750 nM) were introduced, and the mixture was incubated at 4°C for 20 min.

### 4.8 Cell Viability Assay

MCF‐10A cells were seeded at 1 × 10^5^ cells per well and cultured overnight. After washing twice with PBS, the cells were treated with CTDs (500 nM) for different time periods (12 to 36 h). Following incubation, CCK‐8 reagent (10 µL) was added and the cells were incubated for an additional hour. The absorbance at 450 nm was then measured.

### 4.9 Statistical Analysis

All experimental data were obtained from at least five independent replicates and presented as mean ± standard deviation (SD). Data analysis was conducted using GraphPad Prism 8.01. Statistical comparisons between two groups were performed using a one‐way analysis of variance (ANOVA). A p‐value of less than 0.05 was considered statistically significant. The p‐values were indicated as follows: **** for *p* < 0.0001, *** for *p* < 0.001, ** for *p* < 0.01, * for *p* < 0.05, and ns for *p* > 0.05. Flow cytometry data were processed using FlowJo. The signal intensity of confocal images was extracted using Image J software, with a threshold setting of 60. The length distribution of DNA scaffolds was quantified using lmage J and the plugin Skeletonize.

## Author Contributions

X.H. and J.X. performed conceptualization. X.H., J.X., X.G., L.W., and Z.Y. performed methodology. X.H., X.Q., H.L., K.W., X.W., M.S., and J.G. performed Investigation. Y.L. performed project administration. W.G., S.B., C.Y., H.Z. and Y.L. performed supervision. X.H. wrote the original draft. X.H., W.G., S.B., C.Y., H.Z., and Y.L. revised the manuscript.

## Conflicts of Interest

The authors declare no conflicts of interest.

## Supporting information




**Supporting File**: advs73826‐sup‐0001‐SuppMat.docx.

## Data Availability

The data that support the findings of this study are available from the corresponding author upon reasonable request.
