# Supplementary material for: Dynamic Decoration of DNA Scaffolds for High‐Resolution Cancer Cell Subtyping
Source: Adv Sci (Weinh). 2026 Jan 21;13(17):e18307. doi: 10.1002/advs.202518307 (PMC13042676; doi:10.1002/advs.202518307)
Supplement: Supplementary file 1 — Supporting File: advs73826‐sup‐0001‐SuppMat.docx. [file ADVS-13-e18307-s001.docx]

**Supporting Information**

**Dynamic Decoration of DNA Scaffolds for High-resolution Cancer Cell Subtyping**

Xiaolin Hu, Jie Xie, Xinlin Guo, Liangting Wang, Zhengheng Yu, Xiaopei Qiu, Heng Li, Kang Wang, Xiaoxing Wang, Mingxuan Song, Junsong Guo, Wei Gu, Sergio Bernardini, Chaoyong Yang*, Hong Zhang*, Yang Luo*

**Table of Contents**

[Materials and Methods 4](#_Toc216903080)

[Figure S1 The sequence information and predict secondary structure of SDA and SDD tiles 10](#_Toc216903081)

[Figure S2 Optimization of the assembled time of the cy3 and cy5-labeled DNA scaffolds 11](#_Toc216903082)

[Figure S3 Characterization of DNA scaffolds 12](#_Toc216903083)

[Figure S4 Confocal image of DNA scaffolds lacking any strand 13](#_Toc216903084)

[Figure S5 Stability Analysis 14](#_Toc216903085)

[Figure S6 Optimization of the sequence of the DNA tiles 15](#_Toc216903086)

[Figure S7 Feasibility verification of molecular circuits 16](#_Toc216903087)

[Figure S8 The sensitivity curves of input A and input B without molecular circuits 17](#_Toc216903088)

[Figure S9 Dynamic monitoring of the assembled process with both two inputs 18](#_Toc216903089)

[Figure S10 Confocal images of output by AND logic gate without molecular amplifiers 19](#_Toc216903090)

[Figure S11 Confocal images of output by OR logic gate without molecular amplifiers 20](#_Toc216903091)

[Figure S12 The linear curves of (A) input A’ and (B) input B’ at 40 minutes of reaction time 21](#_Toc216903092)

[Figure S13 Confocal images from different channels after a continuous 40-minute reaction with the addition of input A’ and input B’. 22](#_Toc216903093)

[Figure S14 Dynamic monitoring of the DNA scaffold disassembly without toehold domains 23](#_Toc216903094)

[Figure S15 Confocal images of output by NOT gate without molecular amplifiers 24](#_Toc216903095)

[Figure S16 Confocal images of output by NOR gate without molecular amplifiers 25](#_Toc216903096)

[Figure S17 Optimization of the spacer length 26](#_Toc216903097)

[Figure S18 Relative intensity statistics of four leukemia cells treat with the SDA-DRI system 27](#_Toc216903098)

[Figure S19 Confocal images of CEM cells treated with aptamer-modified input strands and random sequences 28](#_Toc216903099)

[Figure S20 The OR-gate-based SDA-DRI for leukemia cell analysis 29](#_Toc216903100)

[Figure S21 The recognition efficiency of the logic device in blood samples 30](#_Toc216903101)

[Figure S22 The response of CTDs to different combination of input strands 31](#_Toc216903102)

[Figure S23 The anchoring efficiency of CTDs to membrane protein 32](#_Toc216903103)

[Figure S24 Cell viability of MCF-10A cells with CTDs at different times 33](#_Toc216903104)

[Figure S25 The relative intensities of different cell lines 34](#_Toc216903105)

[Figure S26 Confocal images of MDA-MB-468 cells treated with aptamer-modified input strands and random sequences 35](#_Toc216903106)

[Figure S27 The NOR-gate-based SDD-TRI system for cancer cell analysis 36](#_Toc216903107)

[Table S1 DNA sequences utilized for the construction of DNA scaffolds. 37](#_Toc216903108)

[Table S2 DNA sequences used for the molecular circuit and logic gates. 38](#_Toc216903109)

[Table S3 DNA sequences used for cell imaging. 40](#_Toc216903110)

# Materials and Methods

**Reagents and materials:** The solutions utilized in the experiments were prepared with ultrapure water (resistance >18 MΩ cm), sourced from a Millipore Milli-Q ultrapure water system (Billerica, USA). All oligonucleotides were synthesized by Tsingke Biotechnology Co., Ltd. (Beijing, China) and purified by high performance liquid chromatography. The detailed sequences were listed in Table S1-S3. RPMI 1640 medium, DMED medium and fetal bovine serum were purchased from Gibco (Shanghai, China). Penicillin streptomycin, 4,6-Diamidino-2-phenylindole (DAPI) and yeast tRNA were acquired from Thermo Fisher Scientific (Shanghai, China). MgCl_2_ was purchased from Sigma-Aldrich (Shanghai, China). 4% paraformaldehyde fixative and Dulbecco's Phosphate Buffered Saline (DPBS) were purchased from Beyotime Biotechnology Co., Ltd (Shanghai, China). DNA markers (25-500 bp), 6 × glycerol gel loading buffer (EDTA xylene cyanol), 4S GelRed (10,000 × in water), ammonium persulfate, 5 × TBE buffer (445 mM Tris base, 445 mM boric acid, 10 mM EDTA, pH 8.0), acrylamide/bisacrylamide 30% solution (29:1), and tetramethylethylenediamine were purchased from Sangon Biotech Co., Ltd (Shanghai, China). HiPure DNA Clean Up Kit was obtained from Magen Biotechnology Co., Ltd. (Guangzhou, China).

**Synthesis of DNA Tiles:** We synthesized two types of DNA scaffolds with distinct initial states, used for self-driven assembly (SDA-Tile) and self-driven disassembly (SDD-Tile) modes. Each type of DNA scaffold was synthesized in Cy5-labeled (SDA-Tile A, SDD-Tile A) and Cy3-labeled (SDA-Tile B, SDD-Tile B) versions, with the detailed scaffold structures shown in Figure S1. Taking the synthesis of SDA-Tile A as an example, A1 (2 μM), Cy5-labeled A2, along with A3 and A4 (4 μM), were mixed in 1 × TAE/Mg²⁺ buffer (40 mM Tris base, 20 mM acetic acid, 2 mM EDTA, and 12.5 mM magnesium acetate, pH 8.0, 50 μL). The solution was then heated to 95℃ using a Mastercycler Gradient Thermal Cycler (Bio-Rad, USA). After heating, the reaction mixture was annealed by cooling at a constant rate to 20℃, with annealing times set at 6, 12, 18, 24, and 30 hours, respectively. Following annealing, the SDA-Tile A was purified using the HiPure DNA Clean Up Kit following the manufacturer’s standard protocol, and stored at a concentration of 1 μM in Elution buffer at -20℃.

**Confocal Fluorescence Microscopy Imaging:** Confocal fluorescence microscopy imaging was conducted with a LSM980 laser scanning confocal microscope (Carl Zeiss, Germany). Unless stated otherwise, the pinhole aperture was adjusted to one Airy unit, and all experiments were performed at ambient temperature. Image acquisition was carried out using either a × 20 objective (Plan-Apochromat × 20/0.8 Air M27) or a × 63 objective (Plan-Apochromat × 63/1.4 oil DIC M27).

**Characterization of DNA scaffolds:** For atomic force microscope, 1 × TAE/Mg^2+^ buffer (20 μL) containing Ni^2+^ (30 mM) was first applied to freshly cleaved mica to shield its negative charge. Subsequently, DNA scaffolds (1 μM, 10 μL) solution was dropped onto the mica surface and allowed to adsorb for 3 to 5 minutes. Afterward, the surface was washed three times with ultrapure water and subsequently dried using compressed air. The prepared samples were imaged in air using the Scan Asyst mode on a Multimode 8 AFM (Bioscope System, Bruker, USA) with a scan rate of 1 Hz and an amplitude setpoint of 300 mV. The obtained images were analyzed and processed using Nanoscope Analysis software. For transmission electron microscope, DNA scaffolds (0.1 μM, 10 μL) was deposited onto a freshly glow-discharged carbon/formvar TEM grid. Prior to the deposition of the solution, the grid was subjected to negative glow discharge for 1 min. After 10 mins of deposition, the grid was treated with a uranyl formate solution (2%) for 15 s. The samples were imaged using an FEI Tecnai G2 120KV microscope (FEI, USA).

**Construction and Validation of Molecular Circuits:** Four molecular amplifiers were constructed for signal amplification, with their structures and detailed reaction processes shown in Figure S6. Among these, molecular amplifiers A to A and B to B were used for AND and NOT logic circuits, while A to B and B to A were employed for OR and NOR logic circuits. The synthesis method for all molecular amplifiers was identical. Taking the molecular amplifier A to A as an example, A4* (2 μM) and input A (2 μM) were mixed in 1 × PBS (50 μL), and hybridized in a SimpliAmp™ PCR instrument (ThermoFisher Scientific, USA) under the following conditions: 95℃ for 5 minutes, followed by a controlled cooling to room temperature at a rate of 0.1℃/s, and then stored at 4℃. For analysis of the molecular circuit reaction process, Cy5-labeled input A* was used to replace tile A.

The molecular circuit reaction process consisted of three steps. Step 1: A3, BHQ-A4, and Cy5-labeled input A* (500 nM) were mixed in 1 × PBS (50 μL), and reacted at 95℃ for 5 minutes, followed by cooling to room temperature at a constant rate of 0.1℃/s; Step 2: Input A (1 μM) was added, and the mixture was incubated at 25℃ for 10 minutes to initiate the first TMSD; Step 3: Molecular amplifier A to A or A to B (1 μM) was added to the system from Step 2, followed by incubation in a metal bath at 25℃ for 10 minutes to initiate the second TMSD. After each step, fluorescence intensity was recorded using an F4700 fluorescence spectrophotometer (Hitachi, Japan).

The gel mixture consisted of distilled water (4 mL), 30% acrylamide/Bis solution (4 mL), 5 × TBE (2 mL), 10% ammonium persulfate (w/v, 100 μL), and tetramethylethylenediamine (10 μL). The gel electrophoresis was performed for 90 minutes at 110 V using a PowerPac^TM^ Universal Power Supply (Bio-Rad, USA). Afterward, the gel was stained with 4SGelRed to visualize the nucleic acids, and imaging was performed using the ChemDoc^MP^ System (Bio-Rad, USA).

**SDA Standard Procedure:** To test the response of SDA to input signals, we used the input A system as an example. A total of SDA-Tile A (200 nM), SDA-Tile B (200 nM), molecular amplifier A to A and B to B (500 nM) were pre-incubated in 1 × TAE/Mg²⁺ buffer (50 μL). Different concentrations of input A or input B (ranging from 50 pM to 2 nM) were added, and the reaction was carried out at 25℃ for 0 to 120 minutes. Fluorescence intensity (λ_ex_ = 550 nm and 649 nm) was measured every 20 minutes using an F4700 fluorescence spectrophotometer for each concentration. For the input concentration of 1 nM, laser confocal microscopy was used to analyze the assembly efficiency from 0 to 100 minutes, with images collected every 20 minutes.

**Construction of SDA-based Logic Gates:** To construct an AND logic gate, SDA-Tile A (200 nM), SDA-Tile B (200 nM), molecular amplifier A to A and B to B (500 nM) were pre-mixed in 1 × TAE/Mg²⁺ buffer (50 μL). The input concentrations were varied as follows: 0 nM input A and 0 nM input B (No input group), 1 nM input A and 0 nM input B (Input A group), 0 nM input A and 1 nM input B (Input B group), and 1 nM input A and 1 nM input B (Input A + B group). Reactions were performed at 25℃ for 100 minutes, after which images were captured using laser confocal microscopy. For the control group without molecular circuits, SDA-Tile (200 nM) and input (1 nM) were mixed, reacted at 25℃ for 100 minutes, and then imaged using confocal microscopy. To construct an OR logic gate, the molecular amplifiers in the above system were replaced with A to B and B to A. The other steps remained the same.

**SDD Standard Procedure:** In the initial state, the SDD-Tile exists as DNA scaffolds. Specifically, A1 and B1 (2 μM), A2, A3’, A4’, B2, B3’, and B4’ (4 μM) were mixed in 1 × TAE/Mg²⁺ buffer (50 μL) to prepare the dual-color DNA scaffolds with toehold sites. The solution was gradually cooled from 95℃ to 20℃ at a constant rate over 30 hours. In a separate system, dual-color DNA scaffolds (200 nM) and molecular amplifiers A to B and B to A (500 nM) were used, and various concentrations of input A and B (ranging from 50 pM to 2 nM) were added. The reaction was carried out at 25℃ for 0 to 60 minutes, and fluorescence intensity was measured using a fluorescence spectrophotometer. For the input concentration of 1 nM, laser confocal microscopy was used to analyze the assembly efficiency from 0 to 40 minutes, with images collected every 10 minutes.

**Construction of SDD-based Logic Gates:** To construct a NOT logic gate, the dual-color DNA scaffolds (200 nM), molecular amplifier A to A and B to B (500 nM) were pre-mixed in 1 × TAE/Mg²⁺ buffer (50 μL). Input concentrations were varied as follows: 0 nM input A’ and 0 nM input B (No input group), 1 nM input A’ and 0 nM input B (Input A group), 0 nM input A’ and 1 nM input B (Input B group), and 1 nM input A’ and 1 nM input B (Input A + B group). The reactions were carried out at 25℃ for 40 minutes, and then imaged using laser confocal microscopy. For the control group without molecular circuits, dual-color DNA scaffolds (200 nM) and input (1 nM) were mixed, reacted at 25℃ for 40 minutes, and then imaged. The relative fluorescence intensity from the confocal images was extracted using ImageJ software. To construct a NOR logic gate, input B was replaced with input B’, and molecular amplifiers were replaced with A to B and B to A in the above system.

**Cell Culture:** All cells were purchased from Type Culture Collection of the Chinese Academy of Sciences (Shanghai, China), and were cultured according to ATCC directions. CEM (human acute T lymphoblastic leukemia cells), Jurkat (human T lymphoblastic leukemia cells), Ramos (human B lymphoma cells), and K562 (human chronic myelogenous leukemia cells) were cultured in RPMI 1640 medium containing 10% fetal bovine serum (FBS) and 1% penicillin-streptomycin, in a 37℃, 5% CO₂ incubator. HepG2 (human liver cancer cells), HeLa (human cervical cancer cells), MCF7 (human breast cancer cells), and MDA-MB468 (triple-negative breast cancer cells) were cultured in DMEM medium containing 10% FBS, streptomycin (100 mg/mL), and penicillin (100 U/mL), at 37℃ and 5% CO₂. MCF-10A (human normal mammary epithelial cells) were maintained in complete MCF-10A medium obtained from Sunncell Biotechnology Co., Ltd. (Wuhan, China). Trypsin was purchased from T&L Biological Technology Co., Ltd. (Beijing, China) and used for cell digestion. For confocal microscopy, cells were initially seeded into confocal dishes and cultured overnight to allow adhesion. For flow cytometry, cells were seeded into 12-well plates and incubated overnight.

**Comparison of Different Imaging Methods:** For Apt-probe methods, CEM cell lines (1 × 10^5^) were washed twice with washing solution (1 mL) containing glucose (4.5 g/L) and MgCl₂ (5 mM) in Dulbecco's phosphate-buffered saline (DPBS) before incubation. A mixture of Cy5-labeled Sgc8c-probe and Cy3-labeled TCO1-probe (200 nM) was incubated with CEM cells in binding buffer (0.1 mg/mL yeast tRNA, 1 mg/mL BSA in washing buffer, 300 μL) at 4℃ for 30 minutes. After incubation, the CEM cells were washed three times with washing buffer (1 mL), followed by centrifugation at 1300 rpm for 3 minutes, and were then resuspended in the same buffer (200 μL).

For Apt-HCR, Sgc8c-S-trigger and TCO1-S-trigger (200 nM) were incubated with CEM cells in binding buffer (300 μL) for 30 minutes. The cells were then washed three times with washing buffer (1 mL), centrifuged at 1300 rpm for 3 minutes, and resuspended in binding buffer (100 μL). Subsequently, Sgc8c-H1, Sgc8c-H2, TCO1-H1, and TCO1-H2 (500 nM) were incubated with the cells at 4℃ for 60 minutes. After three washes with 1 mL washing buffer, the cells were resuspended in washing buffer (200 μL).

For Apt-tile, cells labeled with Sgc8c-S-Input A and TCO1-S-Input B were incubated with SDA-Tile A, SDA-Tile B (500 nM), and molecular amplifiers A to A, and B to B (750 nM) in binding buffer (300 μL) at 4℃ for 100 minutes. Following incubation, the cells were washed three times with washing solution (1 mL) and then resuspended in the same buffer (200 μL).

To assess the stability of long-term imaging, fluorescently labeled cells were incubated at 4℃ for 60, 120, 180, 240, and 300 minutes before imaging with the confocal microscope.

**SDA-DRI Imaging System:** To construct the SDA-DRI system, Sgc8c-S-Input A and TCO1-S-Input B (300 nM) were incubated with K562 or Jurkat, Ramos, CEM cells (1 × 10^5^) in binding buffer (300 μL) at 4℃ for 30 minutes. For the control group, Sgc8c-S-Random and TCO1-S-Random (300 nM) were incubated with CEM cells. After two washes, the cells were centrifuged and resuspended. Subsequently, the cell suspension was incubated at 4°C for 100 minutes after adding SDA-Tile A and SDA-Tile B (500 nM), along with the corresponding molecular amplifiers A to A and B to B (750 nM) for the AND gate, or A to B and B to A for the OR gate. After three washes, the cells were analyzed using flow cytometry and laser confocal microscopy. Data were processed using FlowJo and ImageJ software. To validate the ability of this method to identify specific tumor cell subtypes in a mixed cell population, K562, Jurkat, Ramos, and CEM cells (2.5 × 10^4^) were mixed and processed as described above. Flow cytometry and confocal microscopy were used for analysis.

**Clinical Sample Validation:** For clinical sample simulation, 90 clinical blood samples were collected from Chongqing General Hospital with ethically obtained consent. all samples were from healthy individuals. The study was authorized by the Ethics Committee of Chongqing General Hospital and carried out in accordance with the 1964 Declaration of Helsinki and its subsequent amendments (ethical code KYS2025-047-01). All 90 blood samples were incubated with red blood cell lysis buffer at room temperature for 10 minutes, followed by centrifugation at 2500 rpm for 5 minutes. The precipitated white blood cells were harvested, washed three times with washing buffer, and then resuspended in the same buffer (200 μL). The white blood cells were counted using flow cytometry. Then, each sample of white blood cells were separately spiked with Jurkat, Ramos, or CEM cells at proportions of 0.1%, 0.5%, 1%, 5%, and 10%, respectively. After the treatment with SDA components, all samples were subjected to flow cytometric analysis. Flow cytometry data were analyzed by gating on the target cell population and plotting the fluorescence intensities of the two aptamer-based reporters (Sgc8c and TCO1). The resulting dot plot was divided into four quadrants: Q1 (Sgc8c⁺/TCO1⁻), Q2 (Sgc8c⁺/TCO1⁺), Q3 (Sgc8c⁻/TCO1⁺), and Q4 (Sgc8c⁻/TCO1⁻). The recognition efficiency for each cell line was then calculated as the proportion of cells located in its diagnostic quadrant, normalized to the total population excluding double-negative (Q4) events. The formulas used were:

Equation (1) $Recognition efficiency of Jurkat=\frac{P_{Q1}}{1-P_{Q4}}$

Equation (2) $Recognition efficiency of Ramos=\frac{P_{Q3}}{1-P_{Q4}}$

Equation (3) $Recognition efficiency of CEM=\frac{P_{Q2}}{1-P_{Q4}}$

Where *P* represents the proportion of cancer cell in the indicated quadrants.

**SDD-TRI Imaging System:** For the SDD-TRI system, the following components were mixed to prepare cholesterol-labeled three-color DNA scaffolds (CTDs): Cy5 and cholesterol-labeled SDD-Tile A, Cy3-labeled SDD-Tile B, and FAM-labeled SDD-Tile C (sequences are listed in Table S1; synthesis methods for these tiles are consistent with those for other tiles). Next, EpCAM-S-Input A’, MUC1-S-Input B’, and PTK7-S-Input C’ (300 nM) were incubated with MCF-10A or MCF-7, MDA-MB-468, HepG2, HeLa cells (1 × 10^5^) in binding buffer (300 μL) at 4℃ for 30 minutes, followed by three washes to remove unbound aptamers. For the control group, EpCAM-S-Random, MUC1-S-Random, and PTK7-S-Random (300 nM) were added. Subsequently, CTDs (500 nM) were added and incubated with the aptamer-labeled cells at room temperature for 20 minutes. The cells were washed three times with washing buffer (1 mL) to remove CTDs and reduce background noise. Subsequently, various molecular amplifiers (750 nM) were introduced into the system, and the mixture was incubated at 4℃ for intervals of 5 minutes, ranging from 0 to 30 minutes. After washing three times, the samples were analyzed using flow cytometry. Samples incubated for 20 minutes were used for confocal microscopy imaging to validate the targeting efficiency.

**Cell Viability Assay:** Cell viability was tested using the CCK-8 assay according to the following protocol. First, MCF-10A cells were seeded at 1×10^5^ cells per well in 96-well plates and cultured overnight to allow attachment. After washing twice with PBS, the cells were treated with CTDs (500 nM) for different time periods (12 to 36 hours). Following incubation, CCK-8 reagent (10 μL) was added to each well and the cells were incubated for an additional hour. The absorbance at 450 nm was then measured using a Spark high-throughput microplate reader (Tecan, Switzerland).

**Statistical Analysis:** All experimental data were obtained from at least five independent replicates and presented as mean ± standard deviation (SD). Data analysis was conducted using GraphPad Prism 8.01. Statistical comparisons between two groups were performed using a one-way analysis of variance (ANOVA). A *p*-value of less than 0.05 was considered statistically significant. The *p*-values are indicated as follows: **** for *p* < 0.0001, *** for *p* < 0.001, ** for *p* < 0.01, * for *p* < 0.05, and ns for *p* > 0.05. Flow cytometry data were processed using FlowJo. The signal intensity of confocal images was extracted using Image J software, with a threshold setting of 60. The length distribution of DNA scaffolds was quantified using lmage J and the plugin Skeletonize.


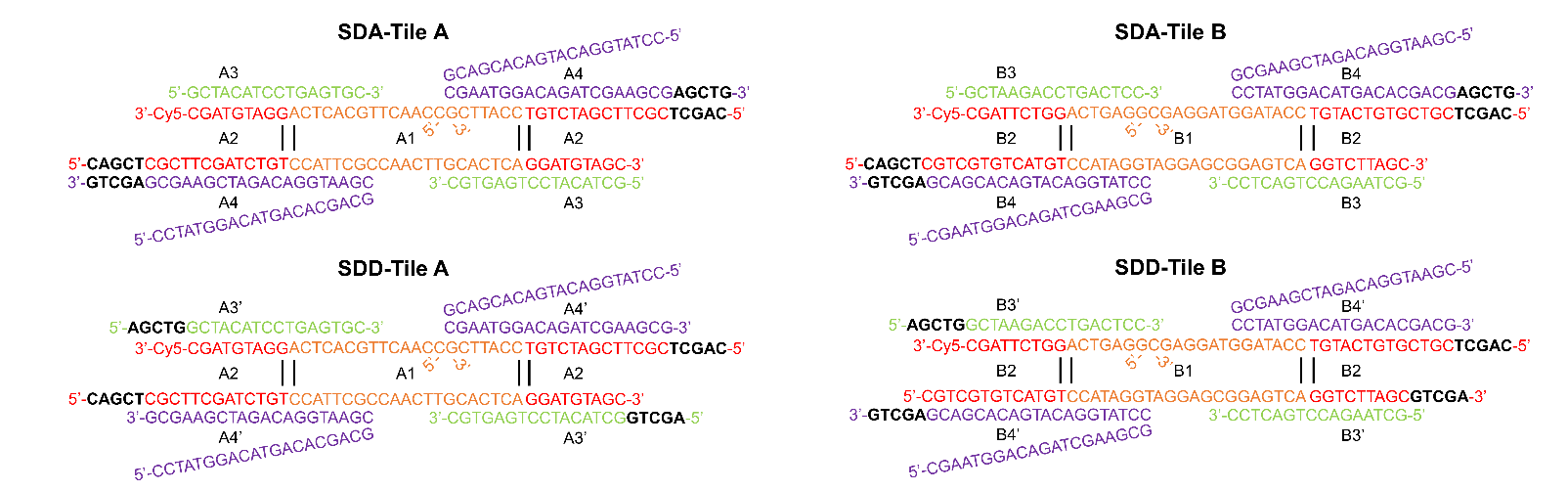


# Figure S1 The sequence information and predict secondary structure of SDA and SDD tiles using NUPACK.


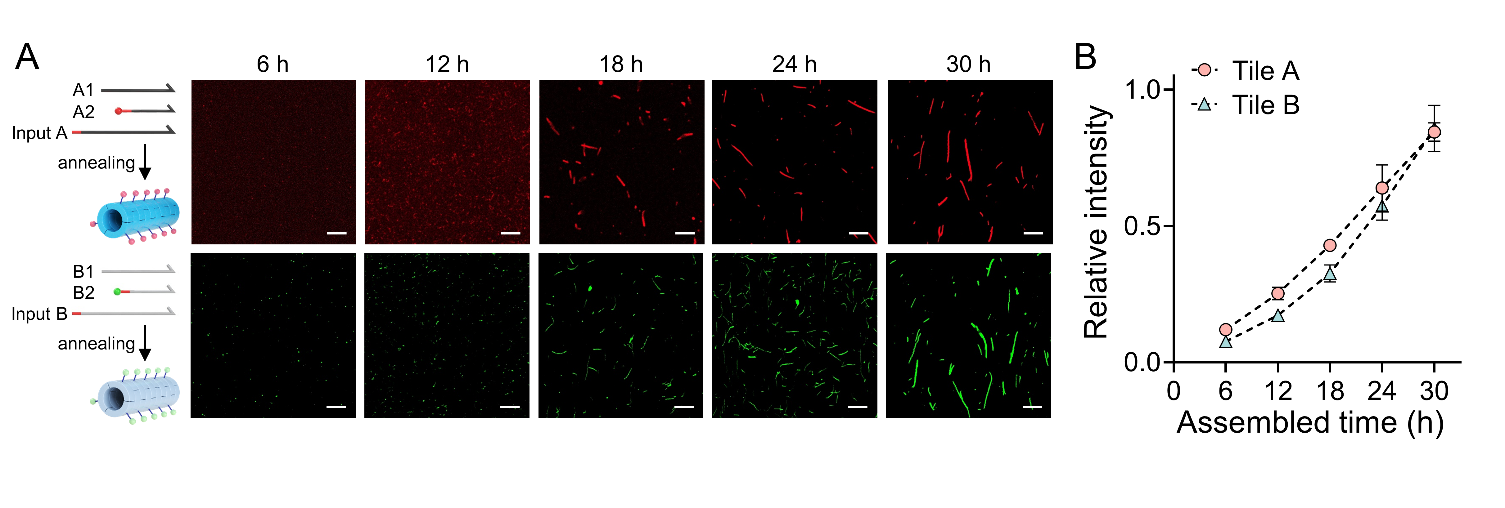


# Figure S2 Optimization of the assembled time of the cy3 and cy5-labeled DNA scaffolds. (A) Confocal image and (B) The relative fluorescence intensity of DNA tiles with different annealing times (6 to 30 hours). Scale bar: 10 μm. Data presented as mean ± SD, n=5.


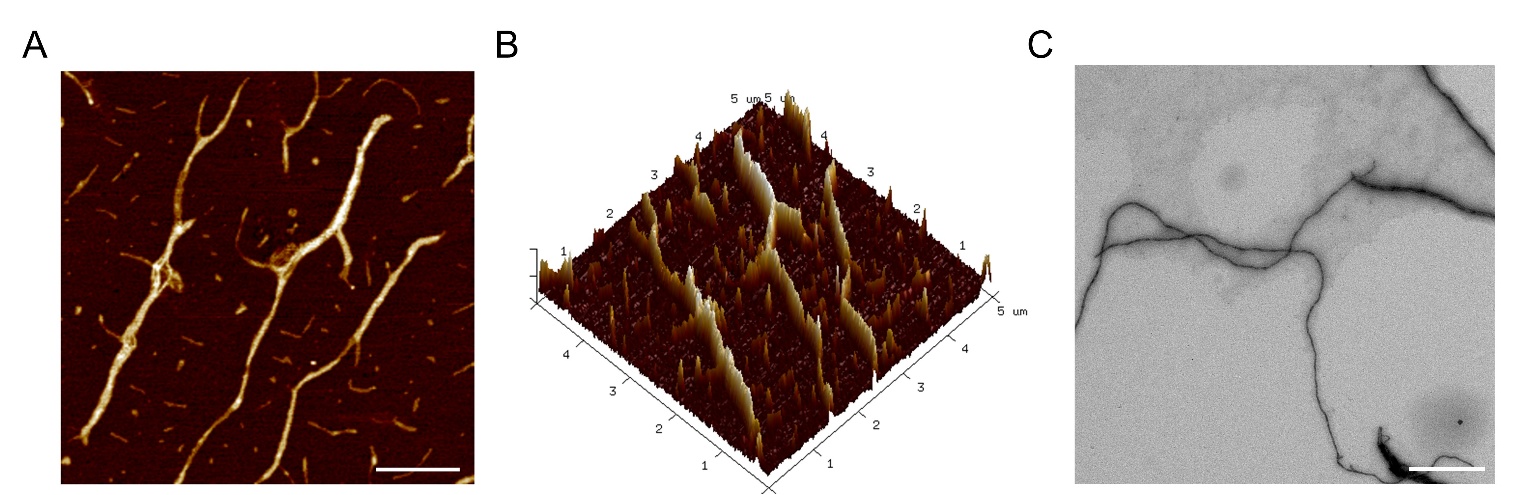


# Figure S3 (A, B) Atomic force microscope and (C) transmission electron microscope characterization of DNA scaffolds after 30 hours of assembly. Scale bar: 1 μm.


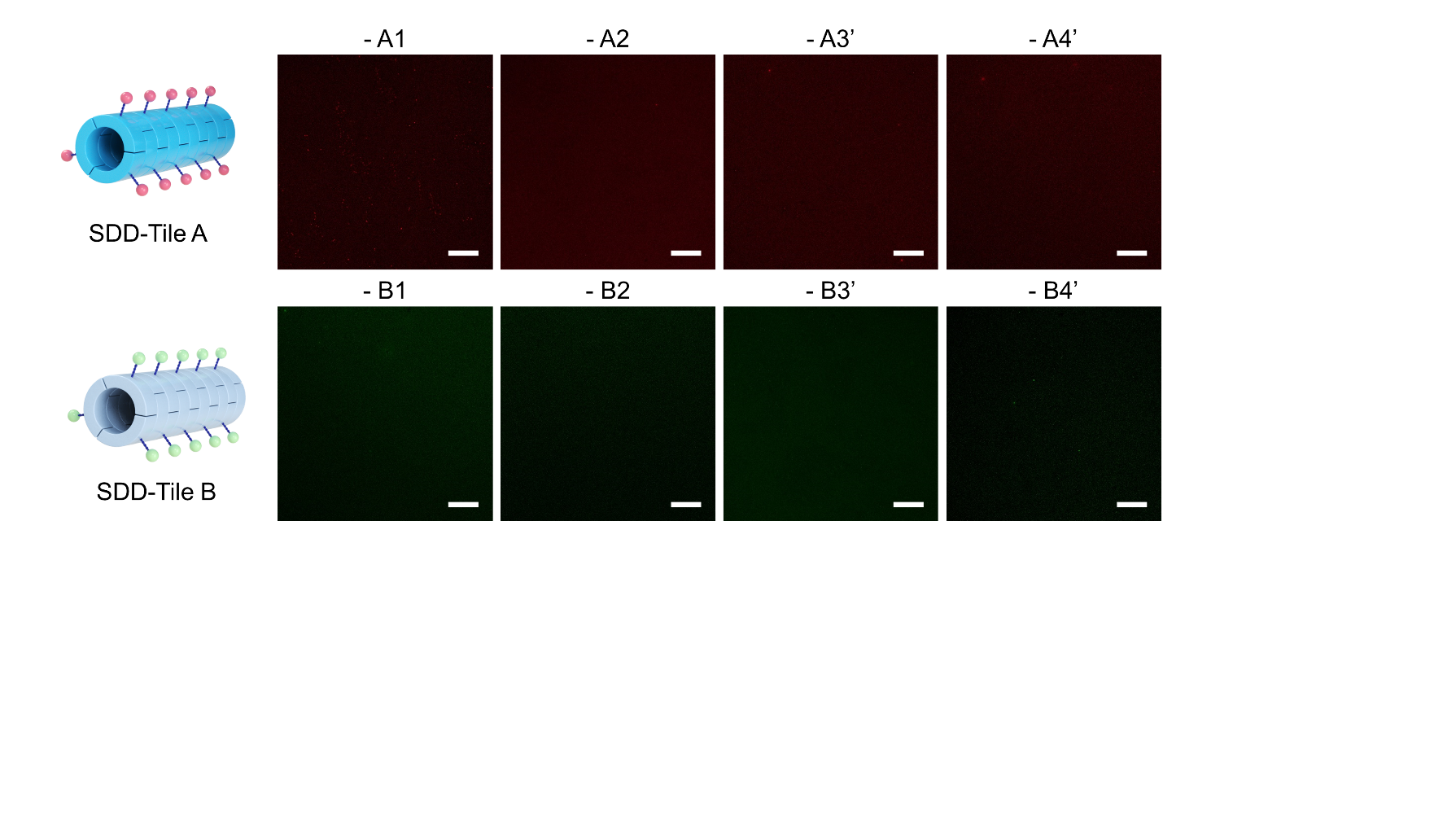


# Figure S4 Confocal image of DNA scaffolds lacking any strand. Scale bar: 10 μm.


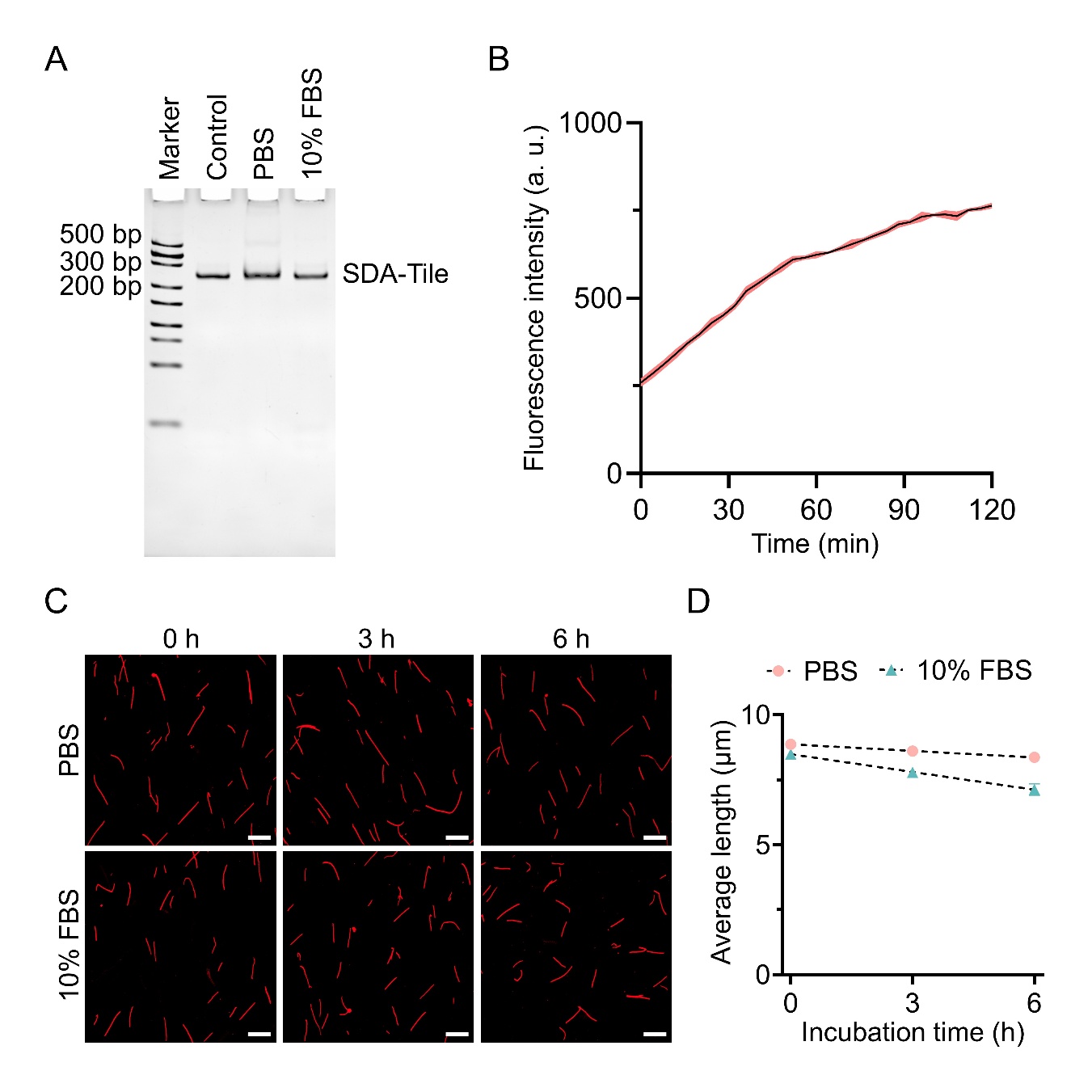


# Figure S5 Stability Analysis. (A) PAGE analysis of SDA-Tile under different physiological conditions. (B) Degradation kinetic curve of SDA-Tile. (C) Confocal images of DNA scaffolds after incubation in PBS and 10% FBS for 0, 3, and 6 hours. (D) Corresponding length distribution of DNA scaffolds under the above conditions. Data presented as mean ± SD, n=5.


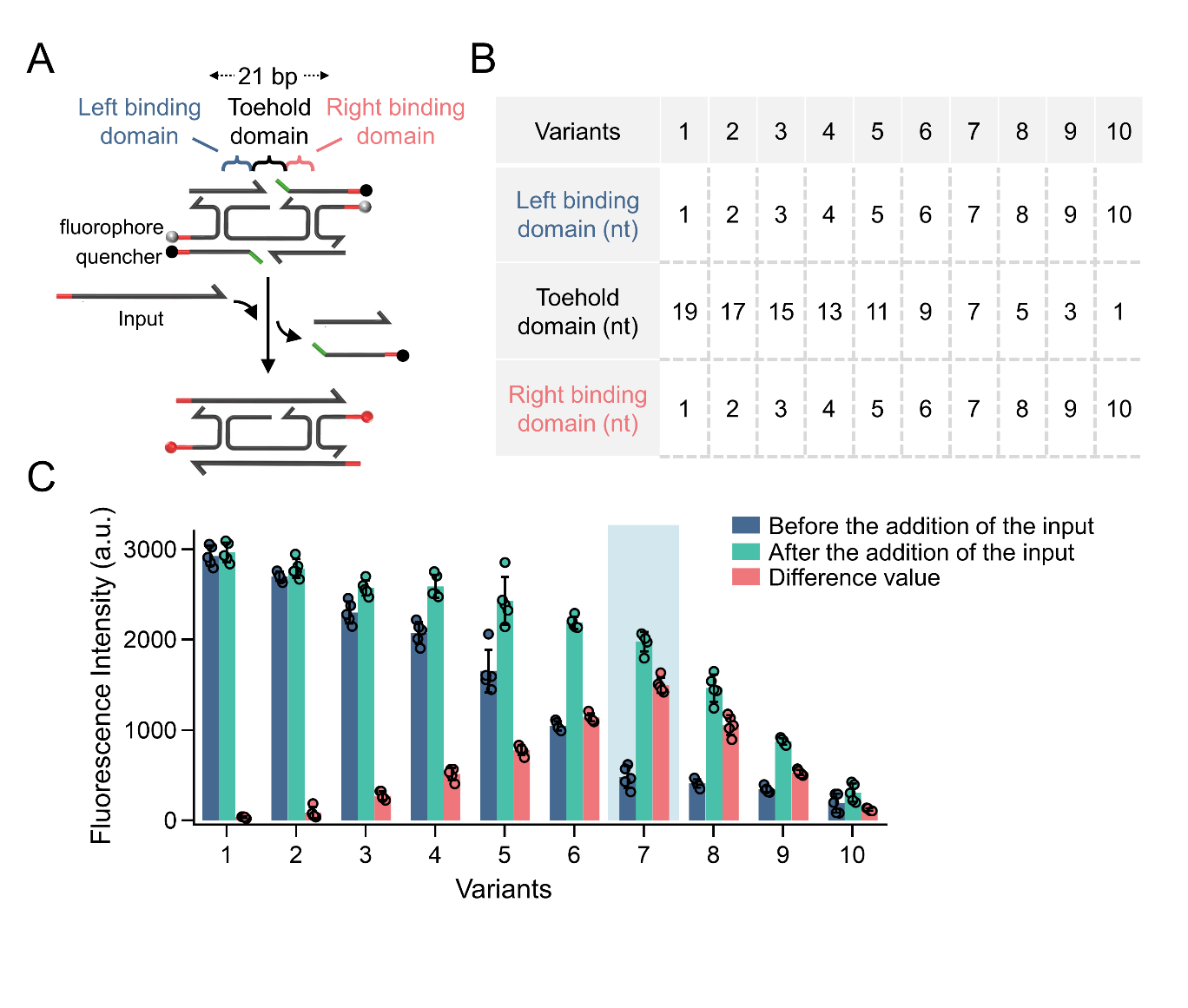


# Figure S6 Optimization of the sequence of the DNA tiles. (A) Scheme of the input-driven toehold-mediate strand displacement (TMSD). The 21-nucleotides TMSD region is divided into the left binding domain, the toehold binding domain, and the right binding domain. The left and right binding domains govern the assembly efficiency of the DNA tiles, while the toehold domain modulates the overall efficiency of TMSD. (B) Table of the 10 variants of DNA tiles which containing different lengths of the left binding domain, toehold domain and right binding domain. (C) The fluorescence intensities of different variants before and after the addition of the input strand. The difference value is calculated as the fluorescence intensity of initial state minus after the displacement reaction. Data presented as mean ± SD, n=5.


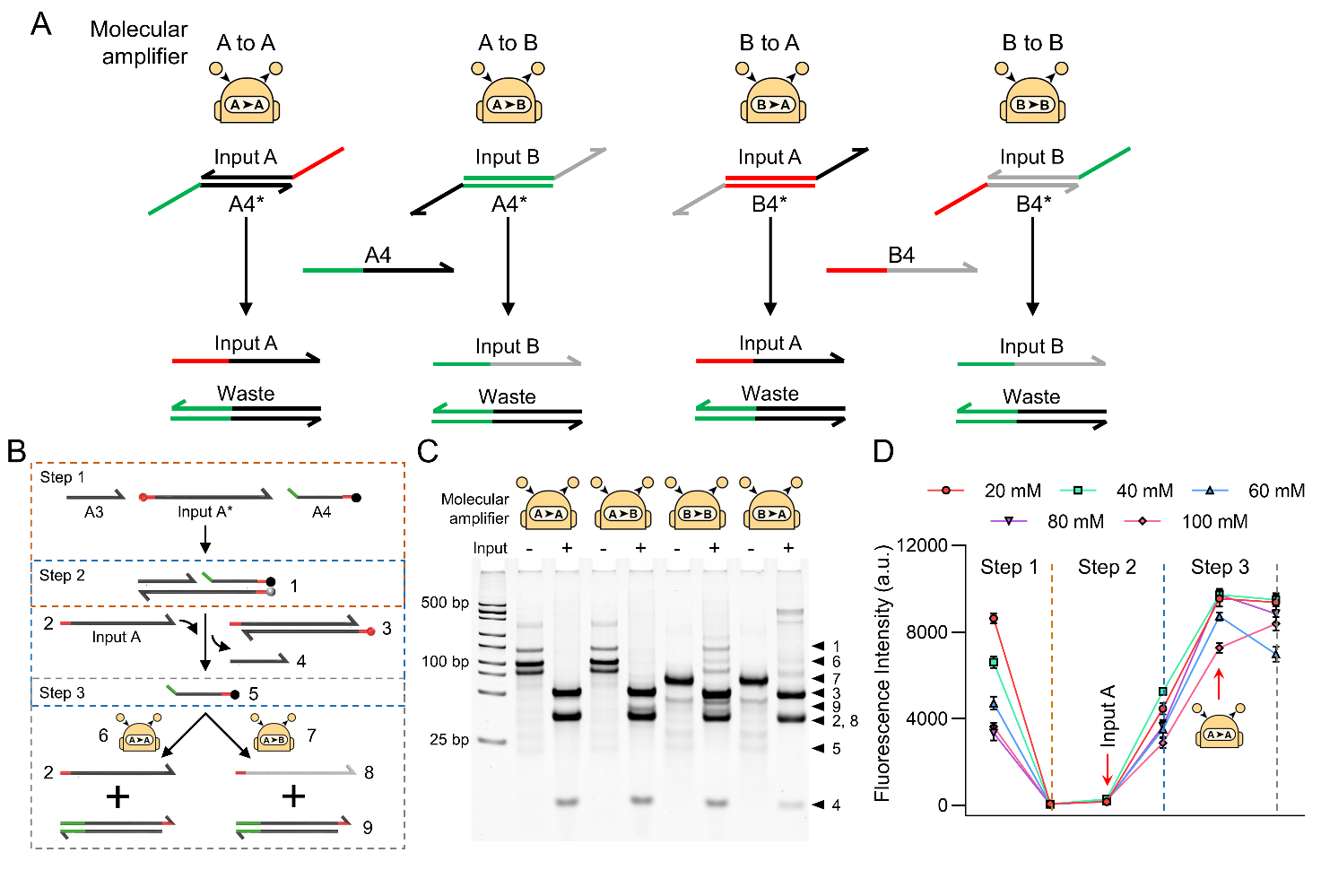


# Figure S7 Feasibility verification of molecular circuits. (A) Working principle of four different molecular circuits. (B) Scheme of TMSD-driven molecular circuit-based signal amplification. The DNA tile was replaced by input A* to simplify the reaction process. The reaction consists of three steps: (1) Cy5-labeled input A* hybridized with A3 and quencher-labeled A4 strand to form a three-strand complex (shown as 1), resulting in fluorescence quenching. (2) Input A (shown as 2) invaded the complex to generate input A-input A* duplex (shown as 3), A3 (shown as 4) and A4 (shown as 5) through TMSD reaction, showing fluorescence recovery. (3) The dissociative A4 underwent a second round of TMSD with the molecular amplifiers A→A (shown as 6) or A→B (shown as 7), regenerating input A or input B (shown as 8) as required, and the waste (shown as 9). (C) PAGE analysis of different four types molecular amplifier (A→A, A→B, B→A, and B→B) in the presence of the corresponding input strand. (D) Fluorescence curves at different stages in S6B for different Mg^2+^ concentrations. Data presented as mean ± SD, n=5.


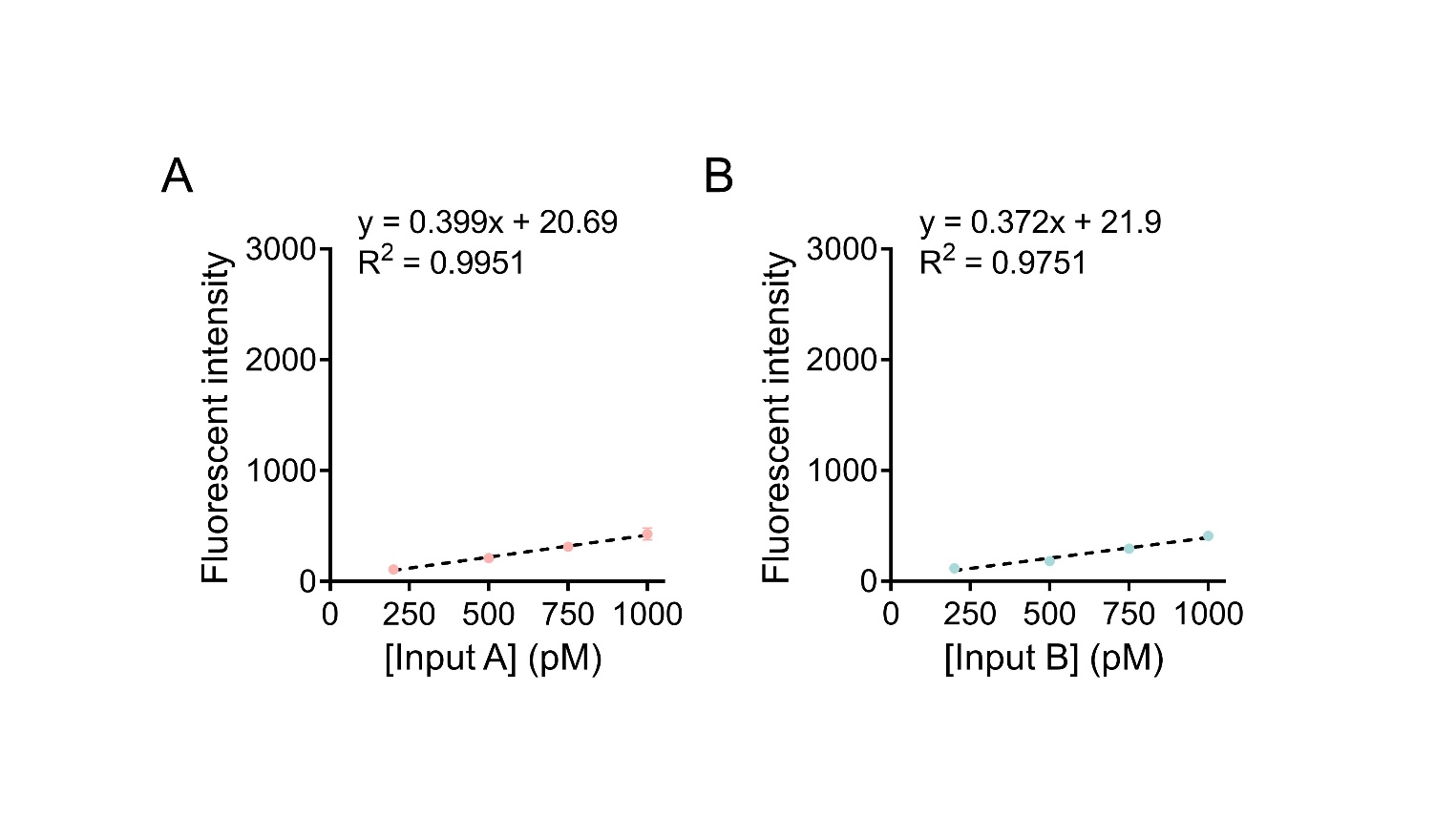


# Figure S8 The sensitivity curves of (A) input A and (B) input B without molecular circuits. Data presented as mean ± SD, n=5.


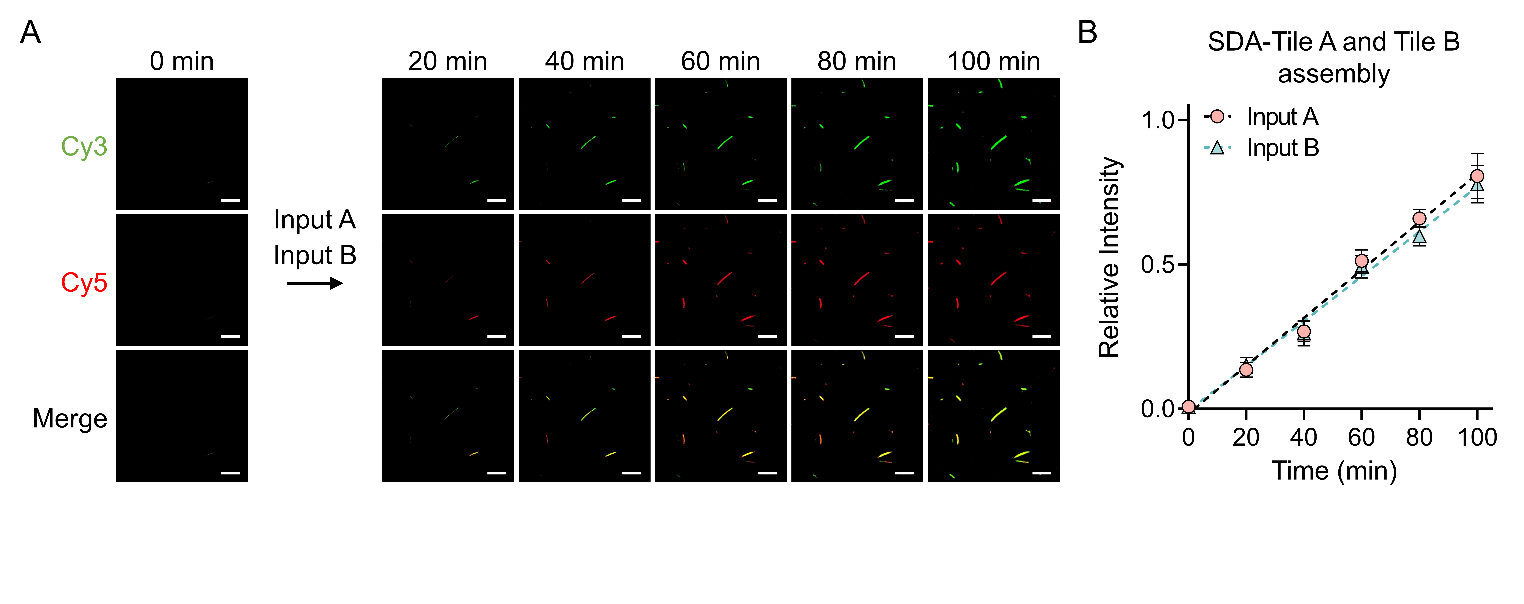


# Figure S9 Dynamic monitoring of the assembled process with both two inputs. (A) Confocal image and (B) relative color intensity after a continuous 100-minute reaction following the simultaneous addition of input A and input B. Scale bar: 10 μm. Data presented as mean ± SD, n=5.


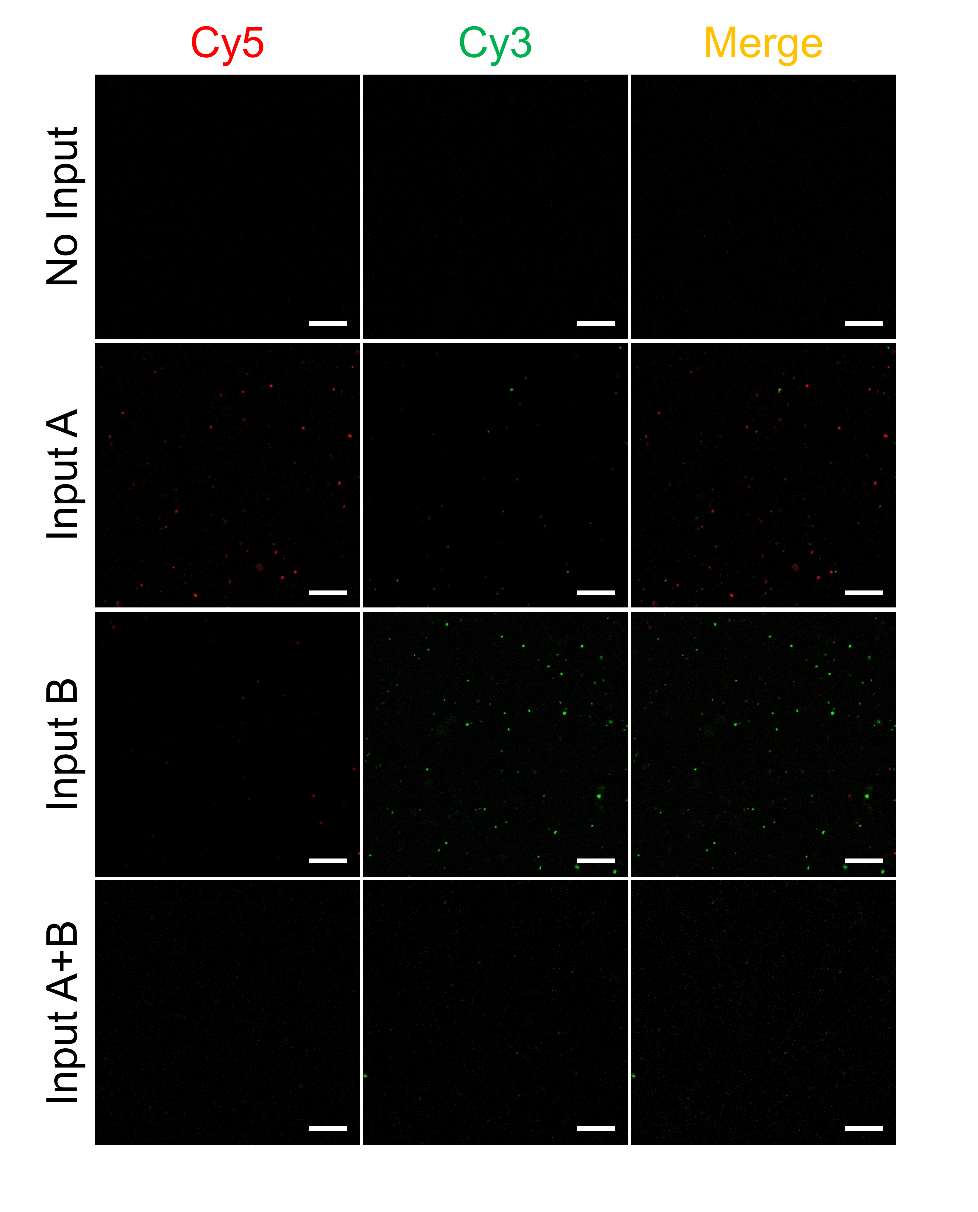


# Figure S10 Confocal images of output by AND logic gate without molecular amplifiers. Scale bar: 10 μm.


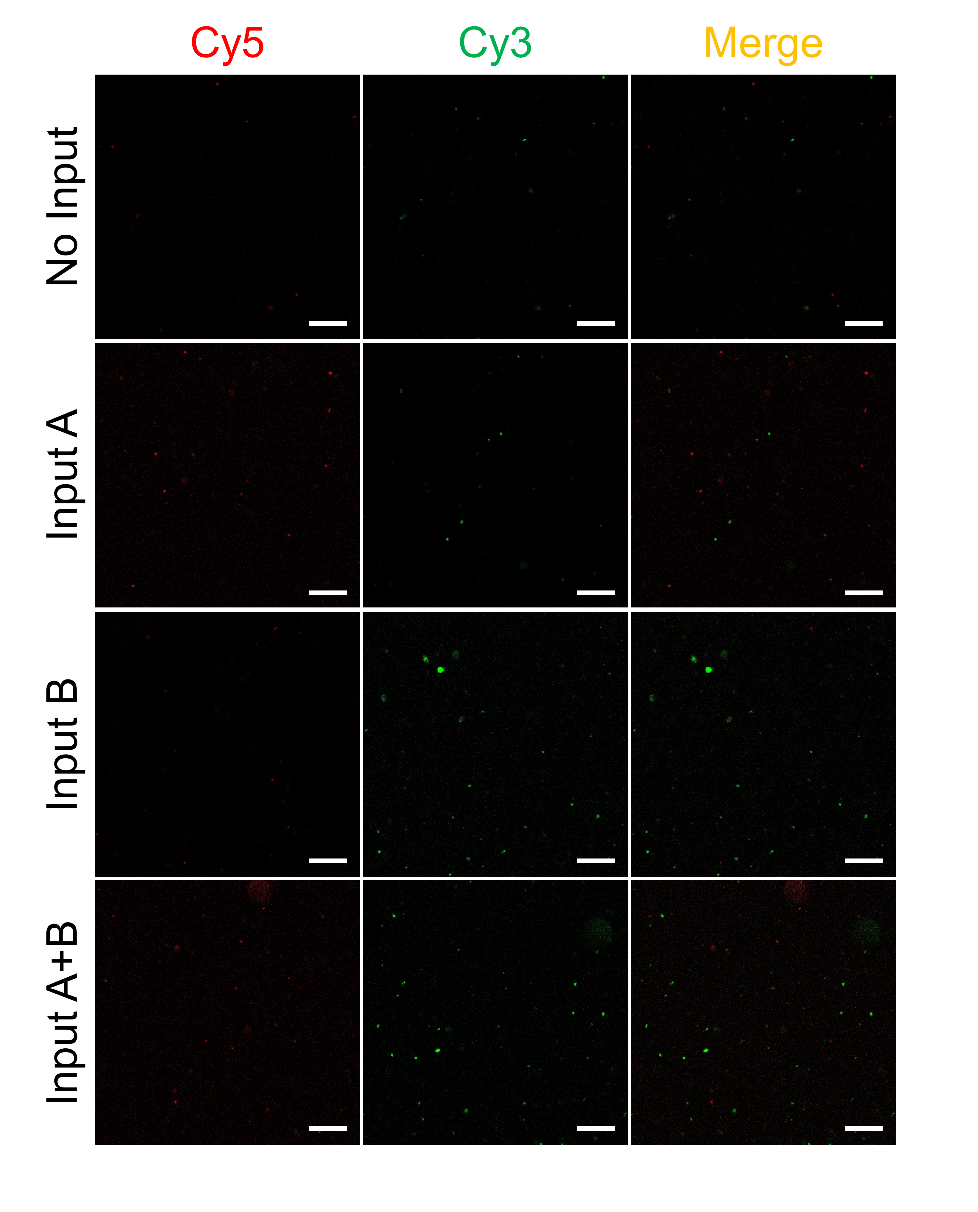


# Figure S11 Confocal images of output by OR logic gate without molecular amplifiers. Scale bar: 10 μm.


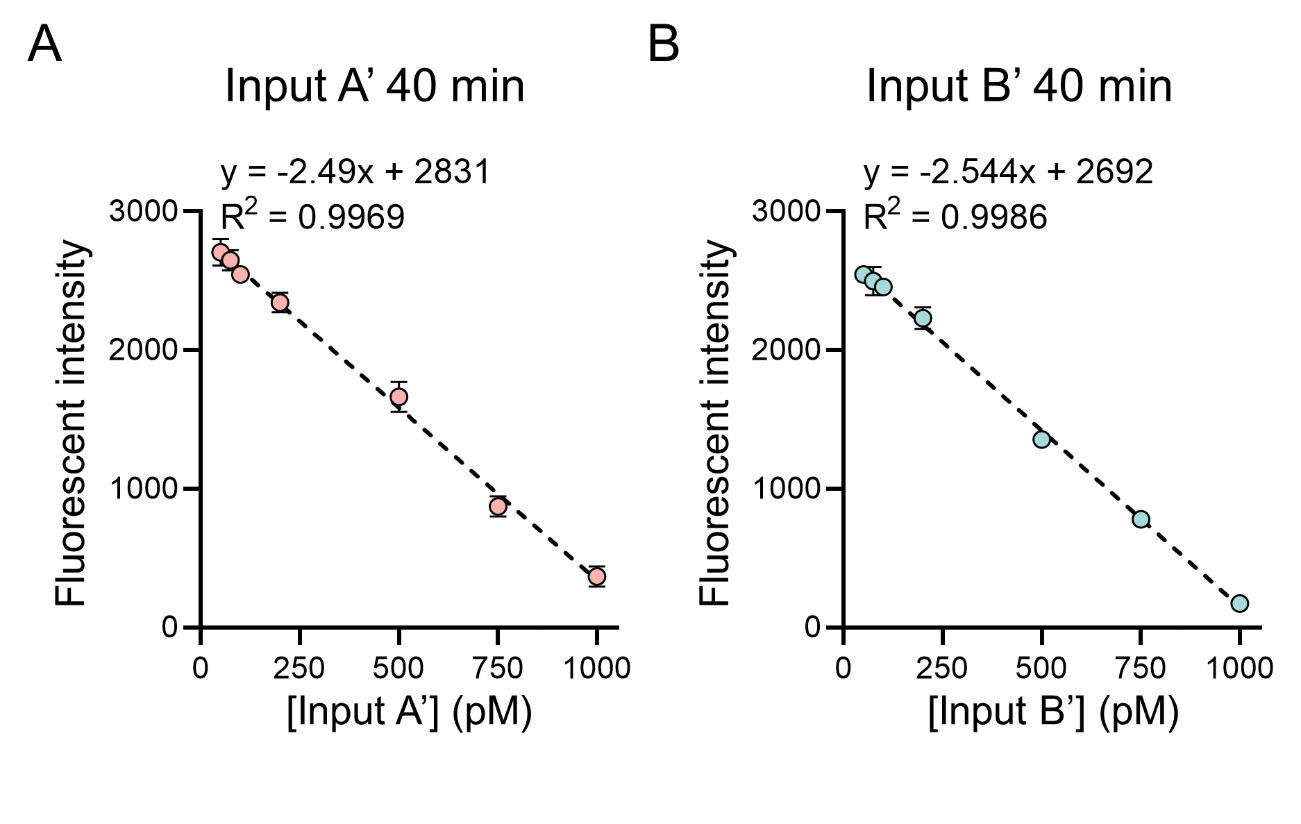


# Figure S12 The linear curves of (A) input A’ and (B) input B’ at 40 minutes of reaction time. Data presented as mean ± SD, n=5.


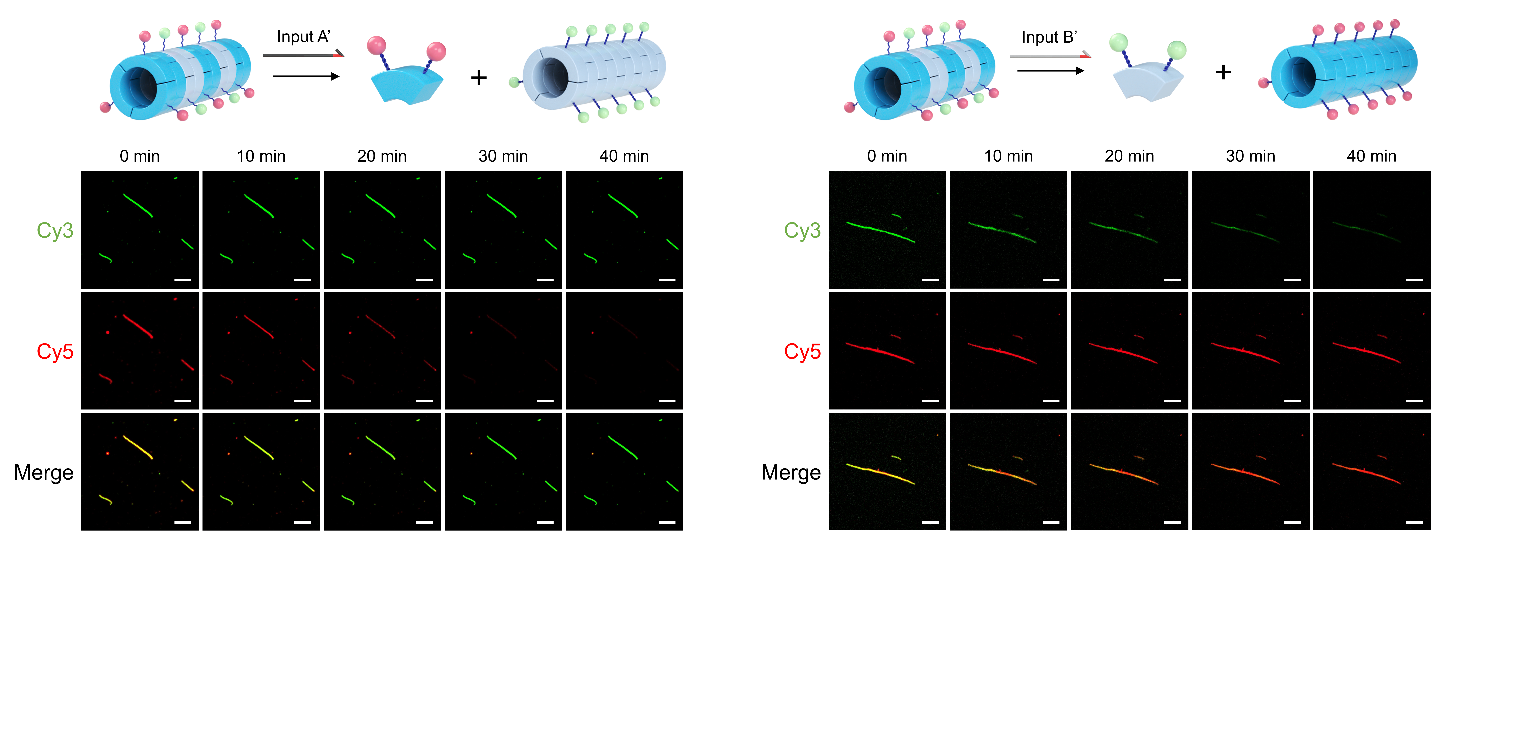


# Figure S13 Confocal images from different channels after a continuous 40-minute reaction with the addition of input A’ and input B’, respectively. Scale bar: 10 μm.


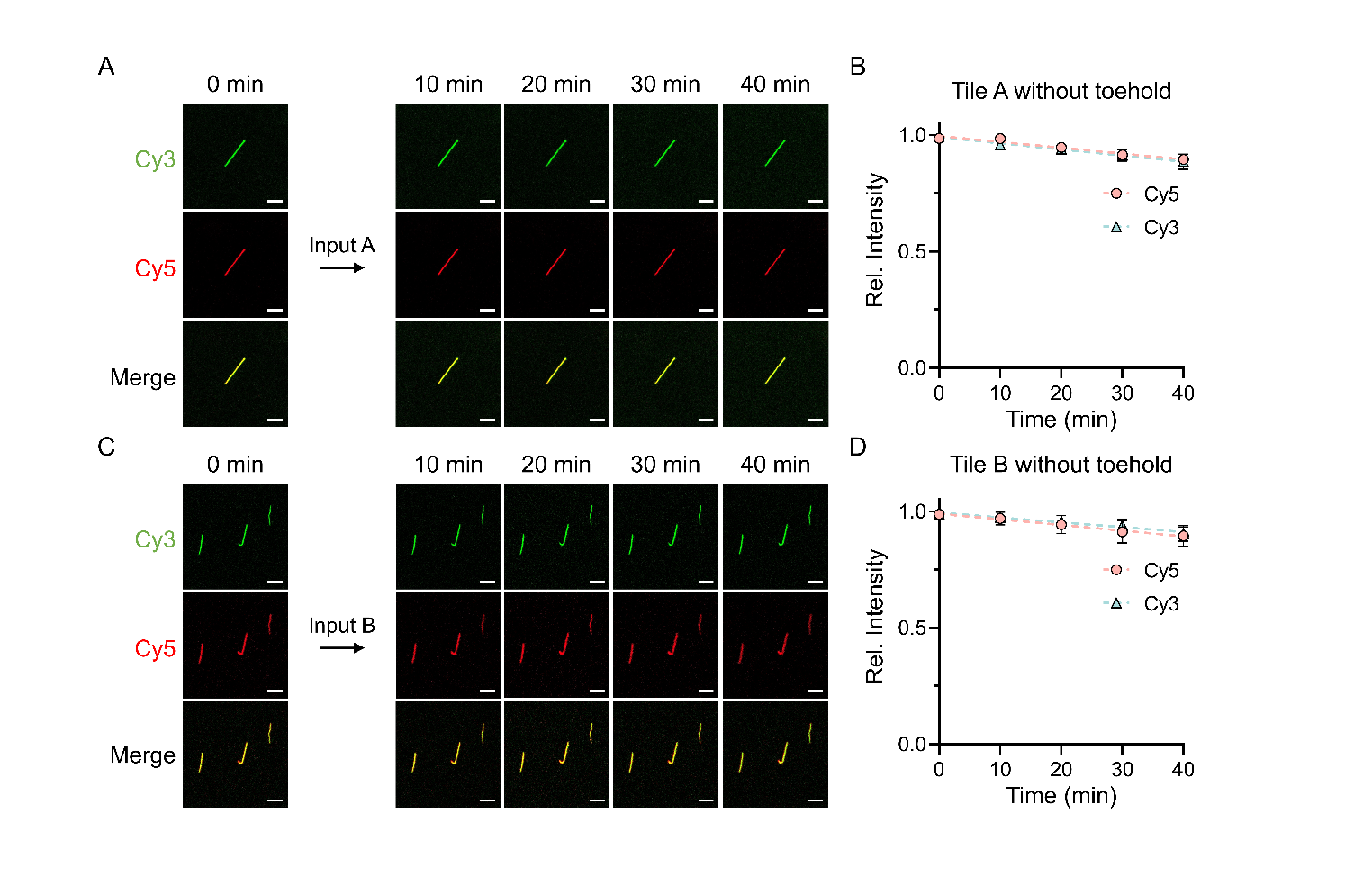


# Figure S14 Dynamic monitoring of the DNA scaffold disassembly without toehold domains. (A) Confocal images and (B) Relative color intensity of DNA scaffolds at different incubation time after the addition of input A. (C) Confocal images and (D) Relative color intensity of DNA scaffolds at different incubation time after the addition of input B. Scale bar: 10 μm. Data presented as mean ± SD, n=5.


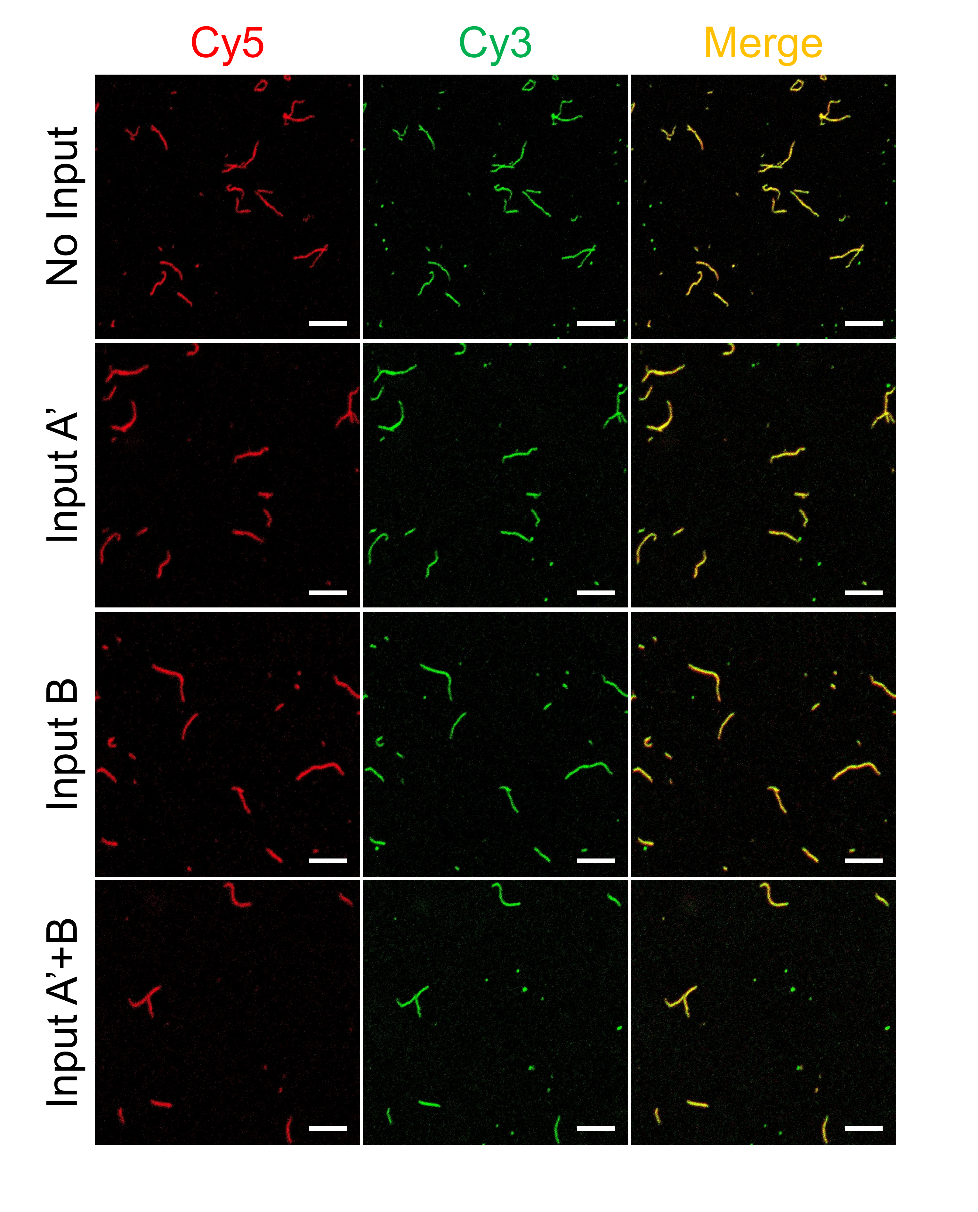


# Figure S15 Confocal images of output by NOT gate without molecular amplifiers. Scale bar: 10 μm.

**
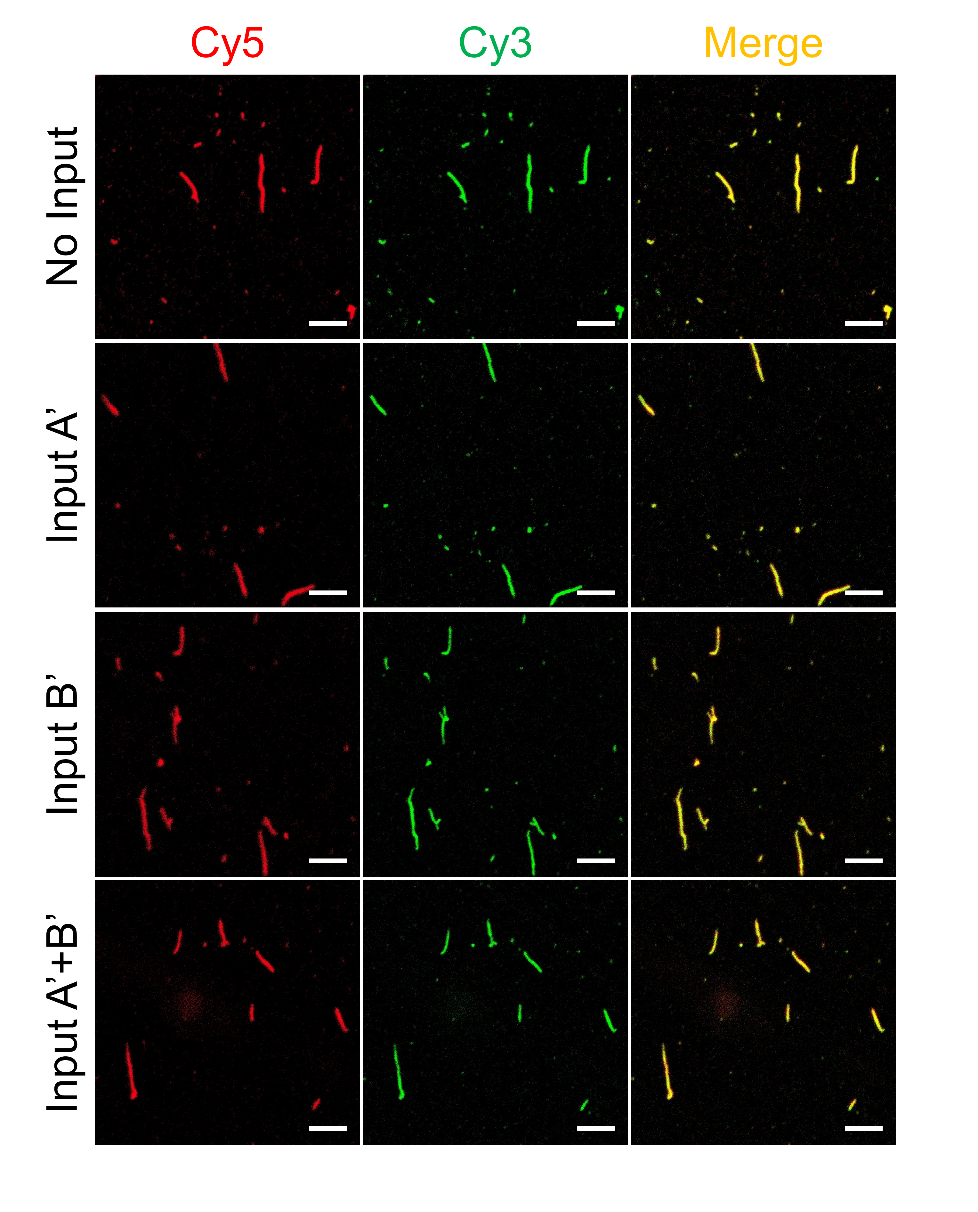
**

# Figure S16 Confocal images of output by NOR gate without molecular amplifiers. Scale bar: 10 μm.


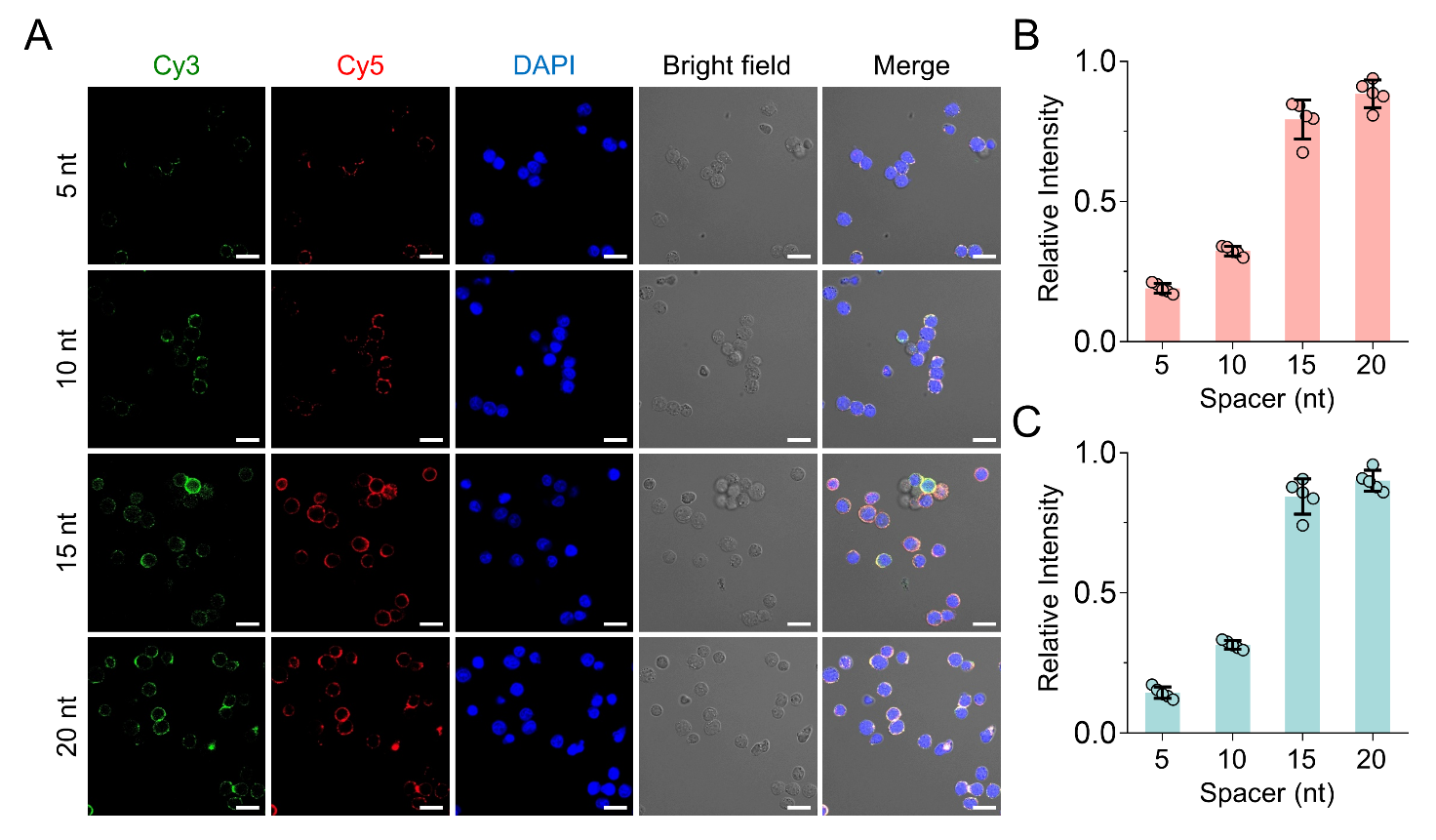


# Figure S17 Optimization of the spacer length. (A) Confocal images of CEM cells incubated with Sgc8c-S-Input A and TCO1-S-Input B, featuring spacers of different nucleotide lengths (5, 10, 15, and 20 nt). Relative fluorescence intensity in the (B) Cy5 and (C) Cy3 channels, as quantified from the confocal images. Data presented as mean ± SD, n=5.


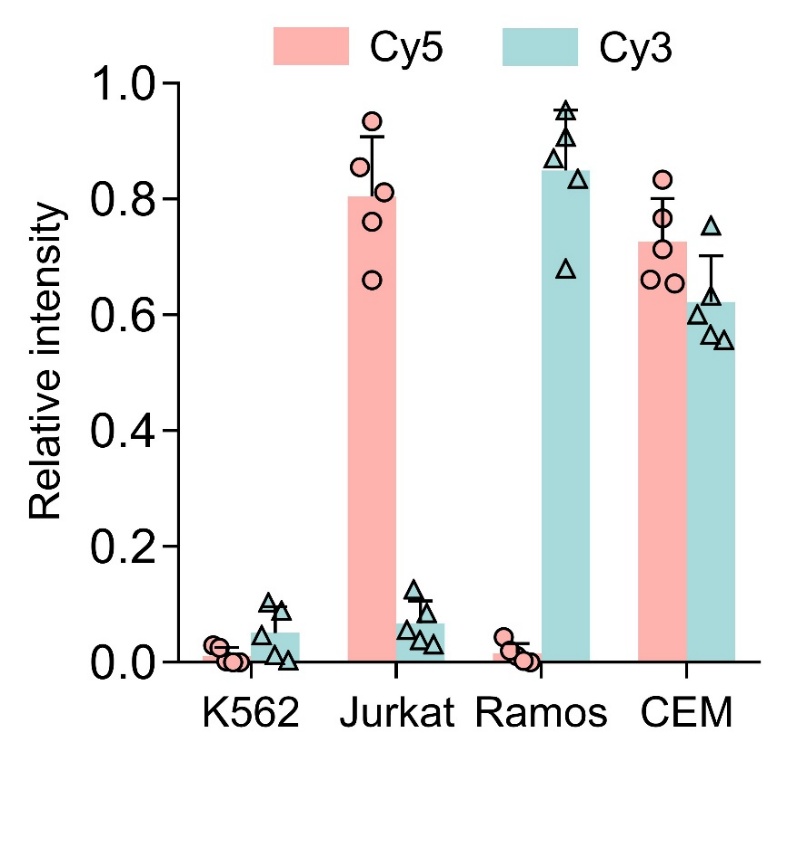


# Figure S18 Relative intensity statistics of four leukemia cells treat with the SDA-DRI system. Data presented as mean ± SD, n=5.


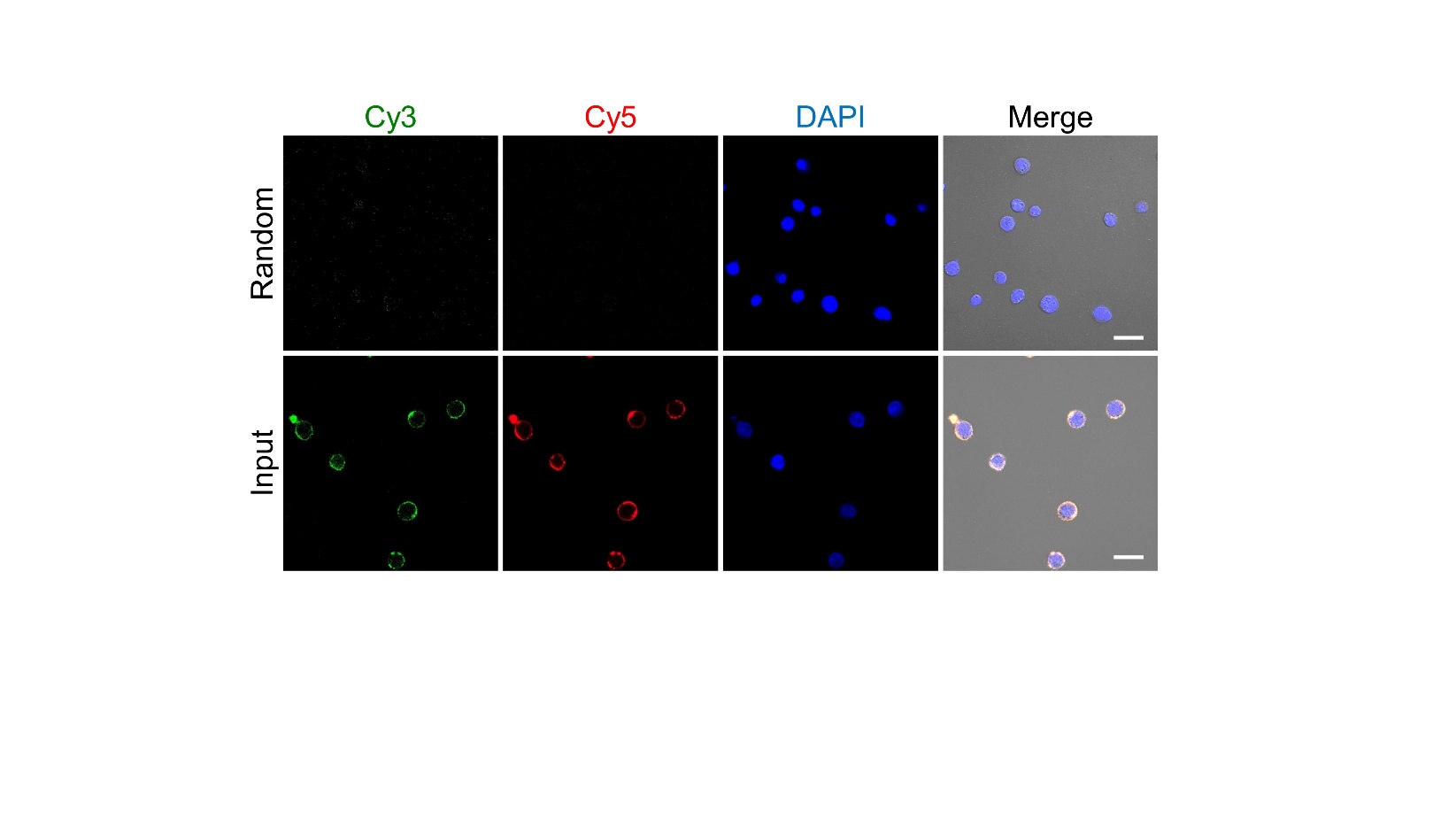


# Figure S19 Confocal images of CEM cells treated with aptamer-modified input strands and random sequences. Scale bar: 20 μm.


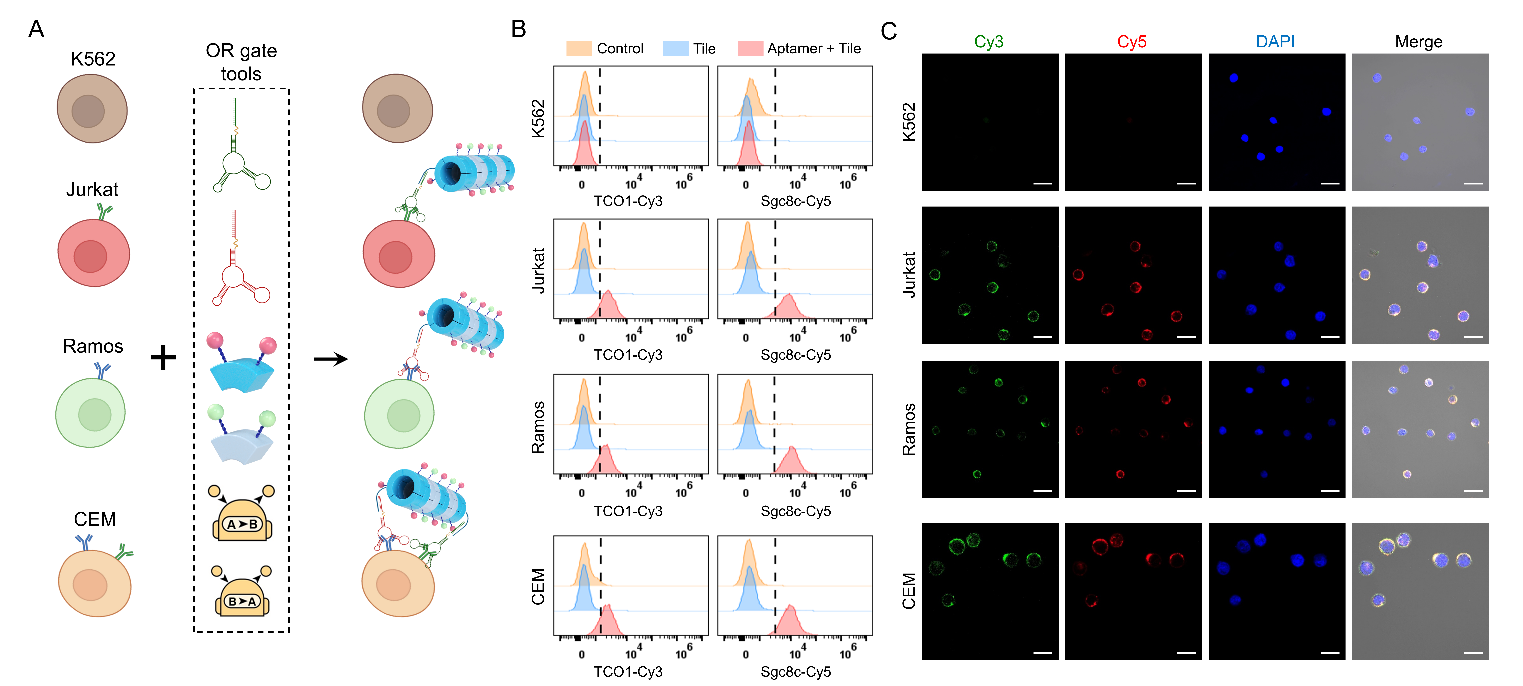


# Figure S20 The OR-gate-based SDA-DRI for leukemia cell analysis. (A) The principle of OR gate-based SDA-DRI system. (B) Flow cytometry analysis for four different cells. (C) Confocal images of four different cell lines with OR gate-based SDA-DRI system. Scale bar: 10 μm.


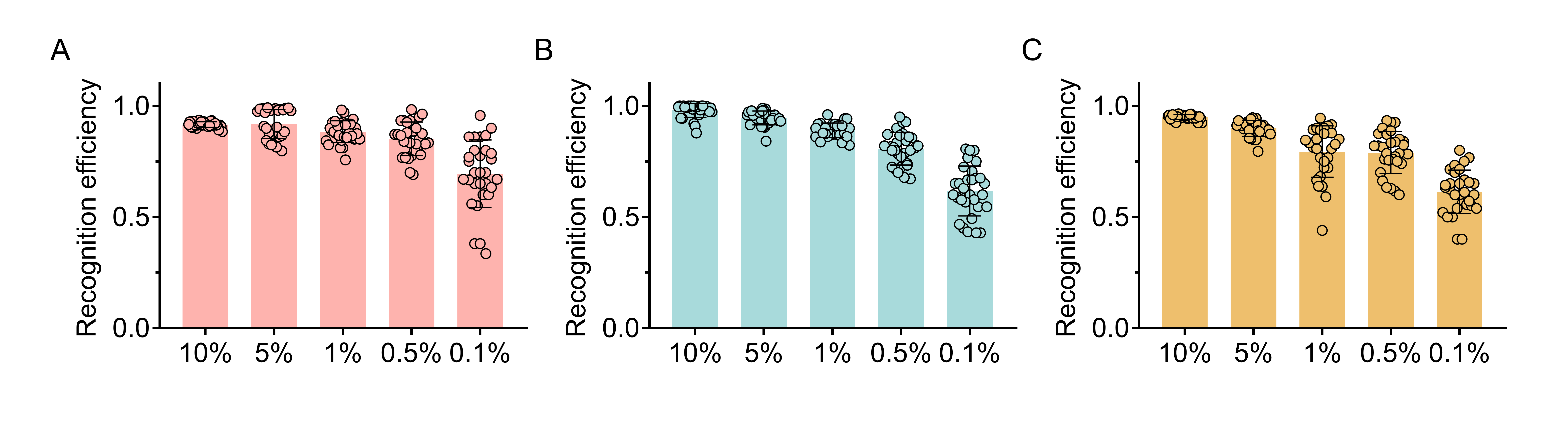


# Figure S21 The recognition efficiency of the logic device in blood samples with different proportions of cancer cells present. (A) Jurkat, (B) Ramos, and (C) CEM. The recognition efficiency was calculated as described in the Materials and Methods section. Data presented as mean ± SD, n=30.


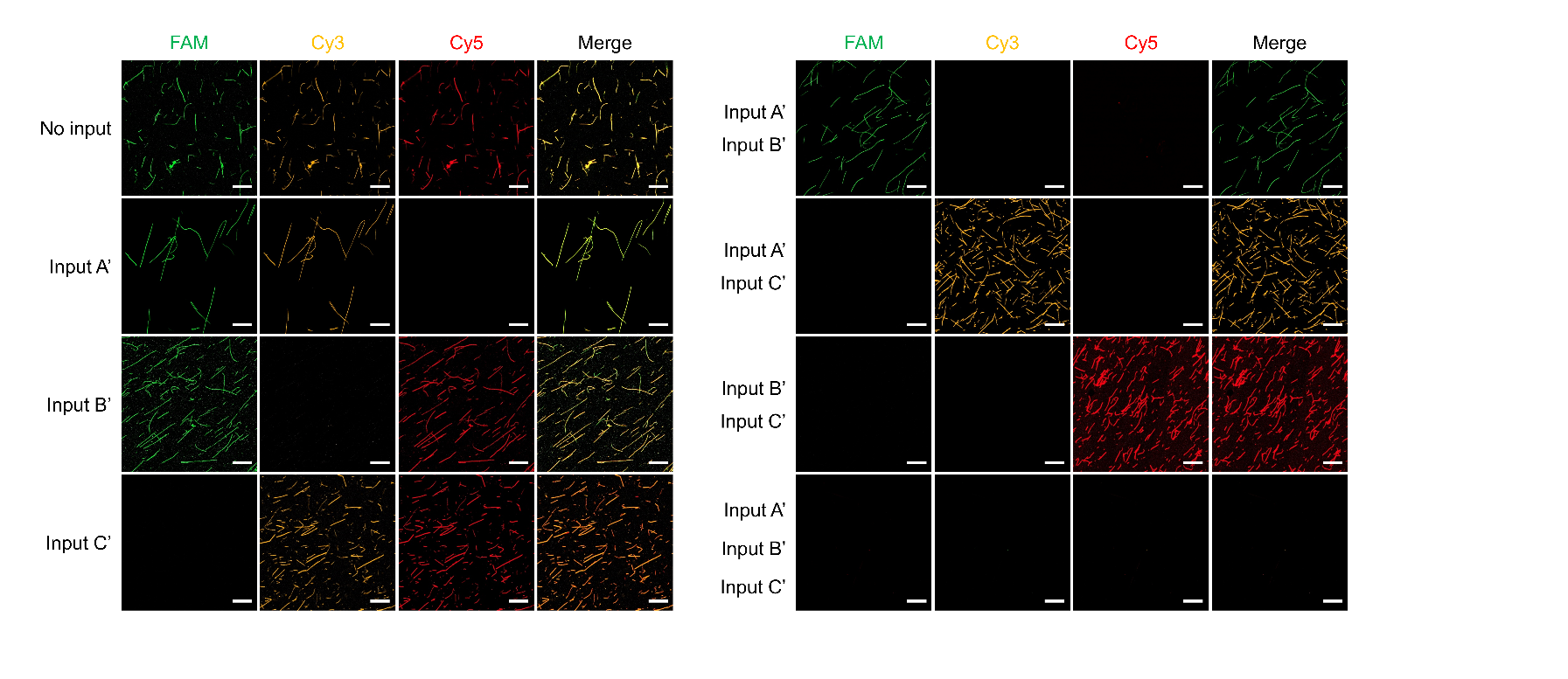


# Figure S22 The response of CTDs to different combination of input strands. Scale bar: 10 μm.


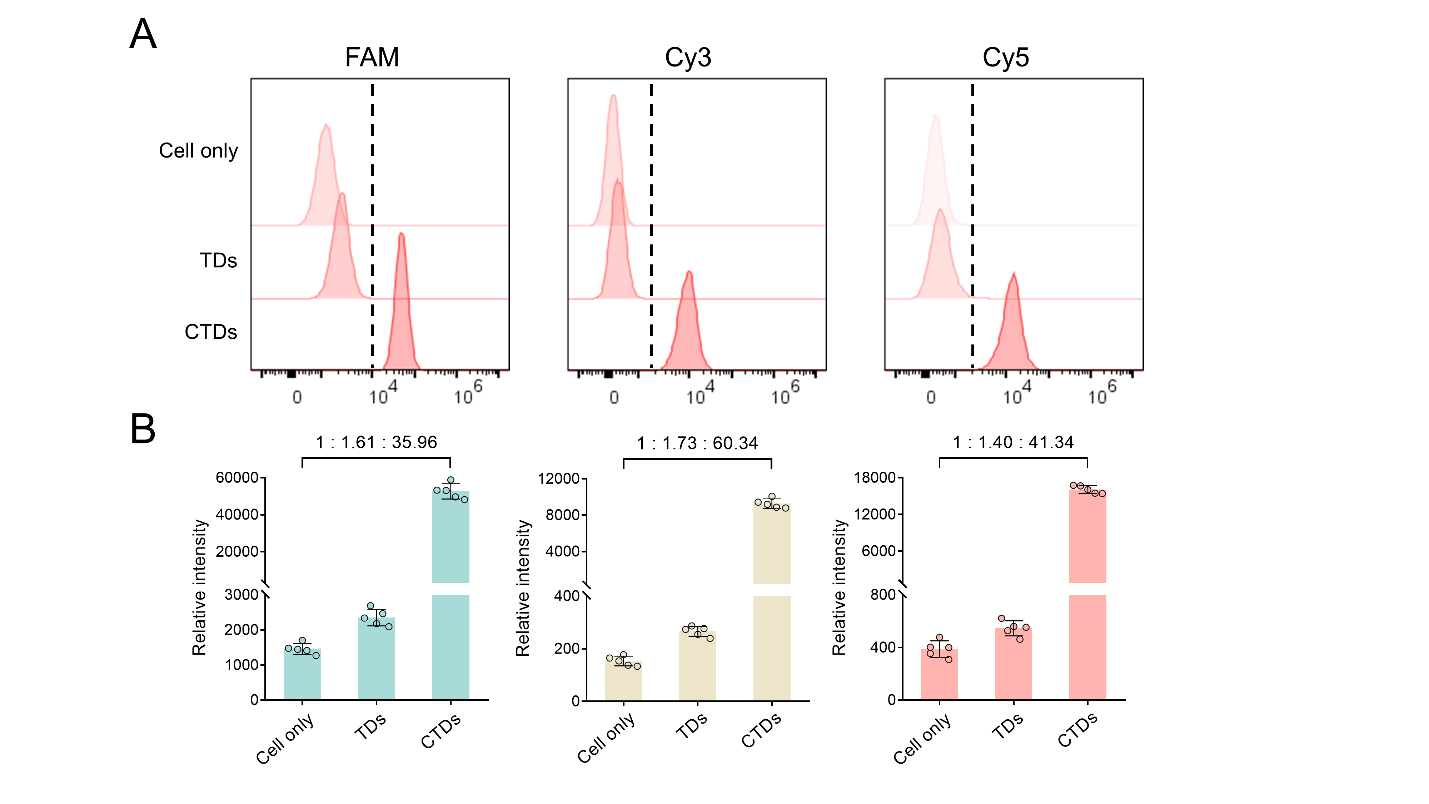


# Figure S23 The anchoring efficiency of CTDs to membrane protein. (A) The flow cytometry analysis and (B) relative intensity statistics of MCF-10A with TDs and CTDs labeling, respectively. Data presented as mean ± SD, n=5.


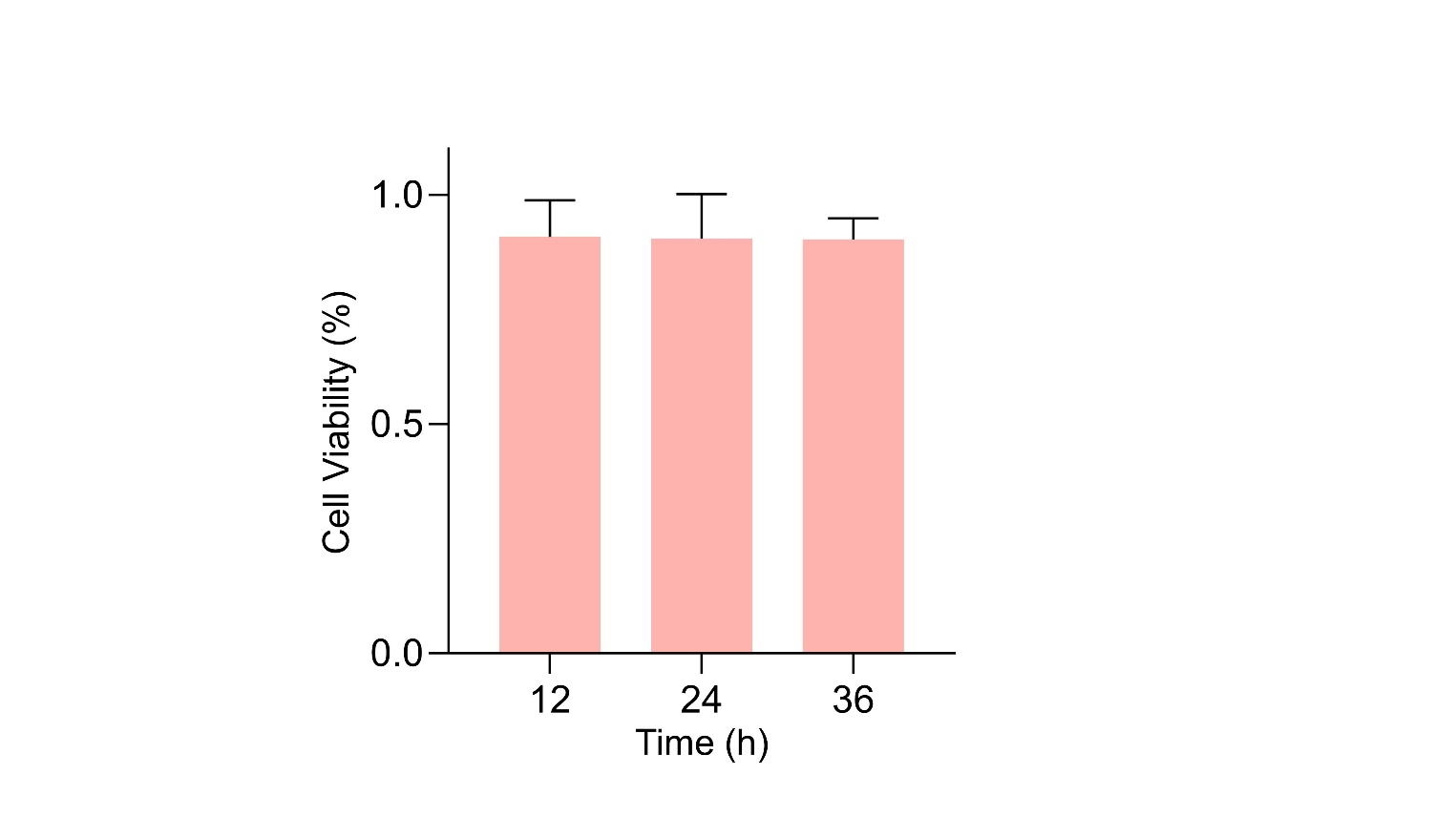


# Figure S24 Cell viability of MCF-10A cells with CTDs at different times. Data presented as mean ± SD, n=5.


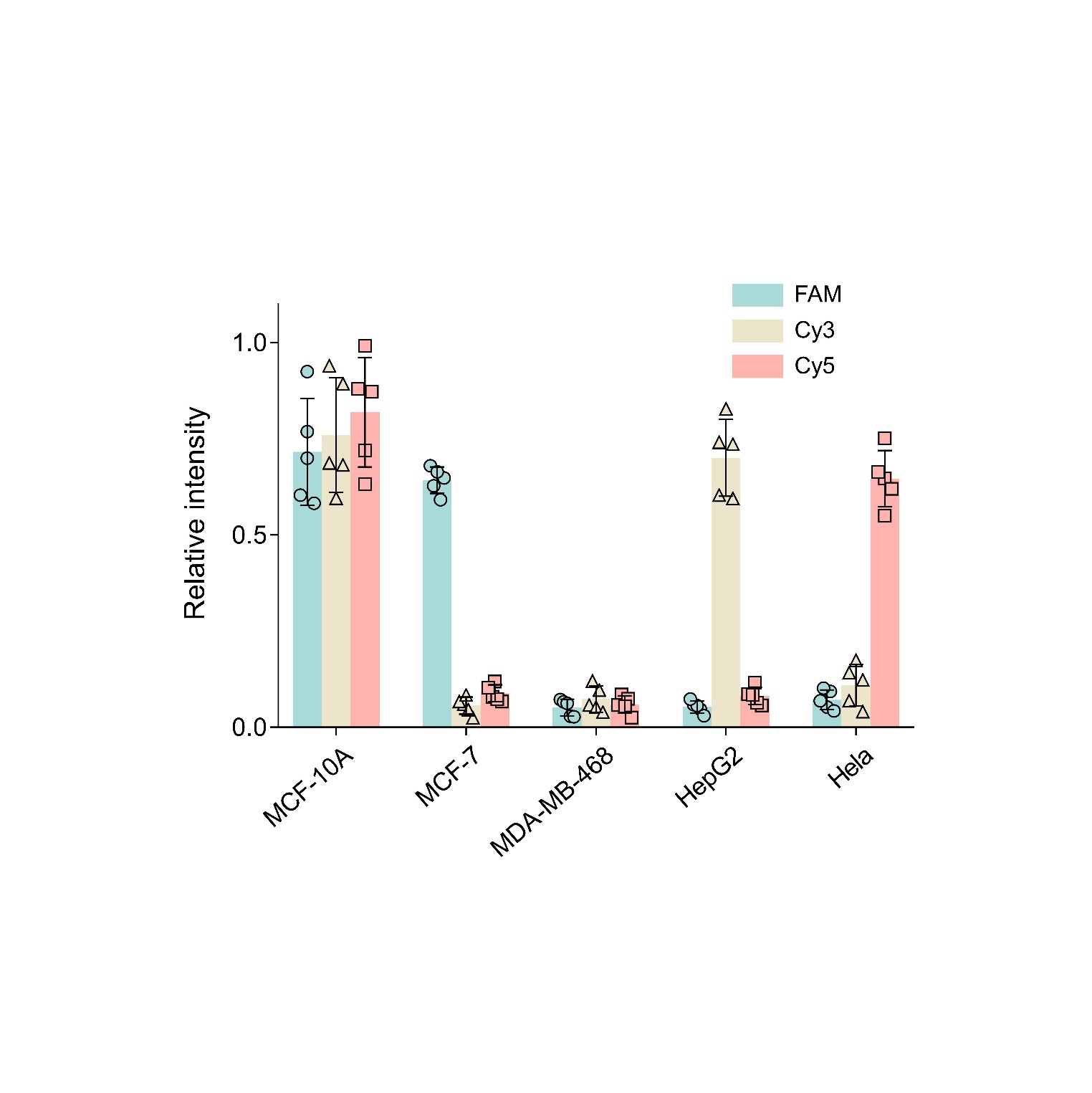


# Figure S25 The relative intensities of different cell lines corresponding to the confocal images in Figure 6F. Data presented as mean ± SD, n=5.


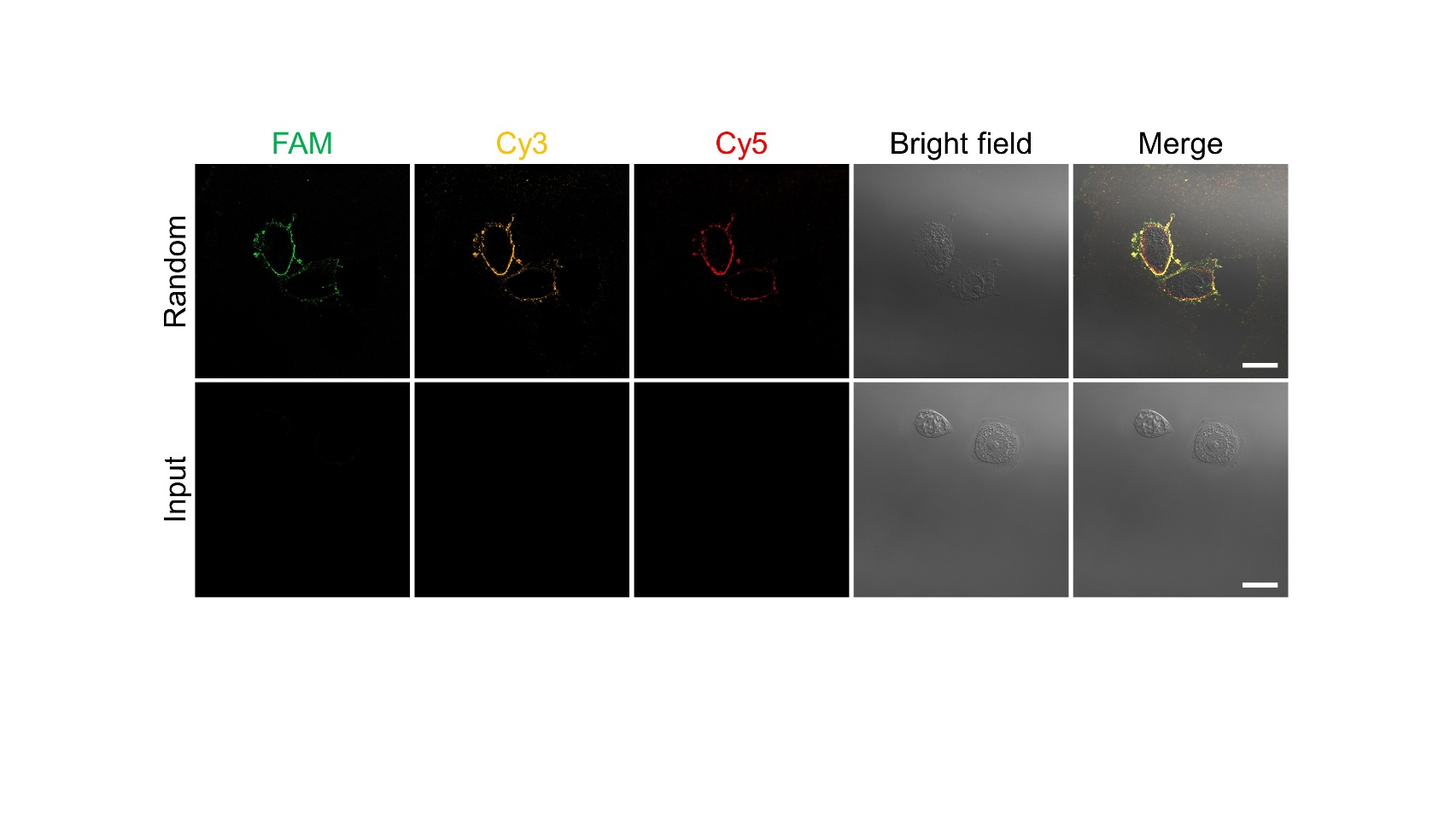


# Figure S26 Confocal images of MDA-MB-468 cells treated with aptamer-modified input strands and random sequences. Scale bar: 10 μm.


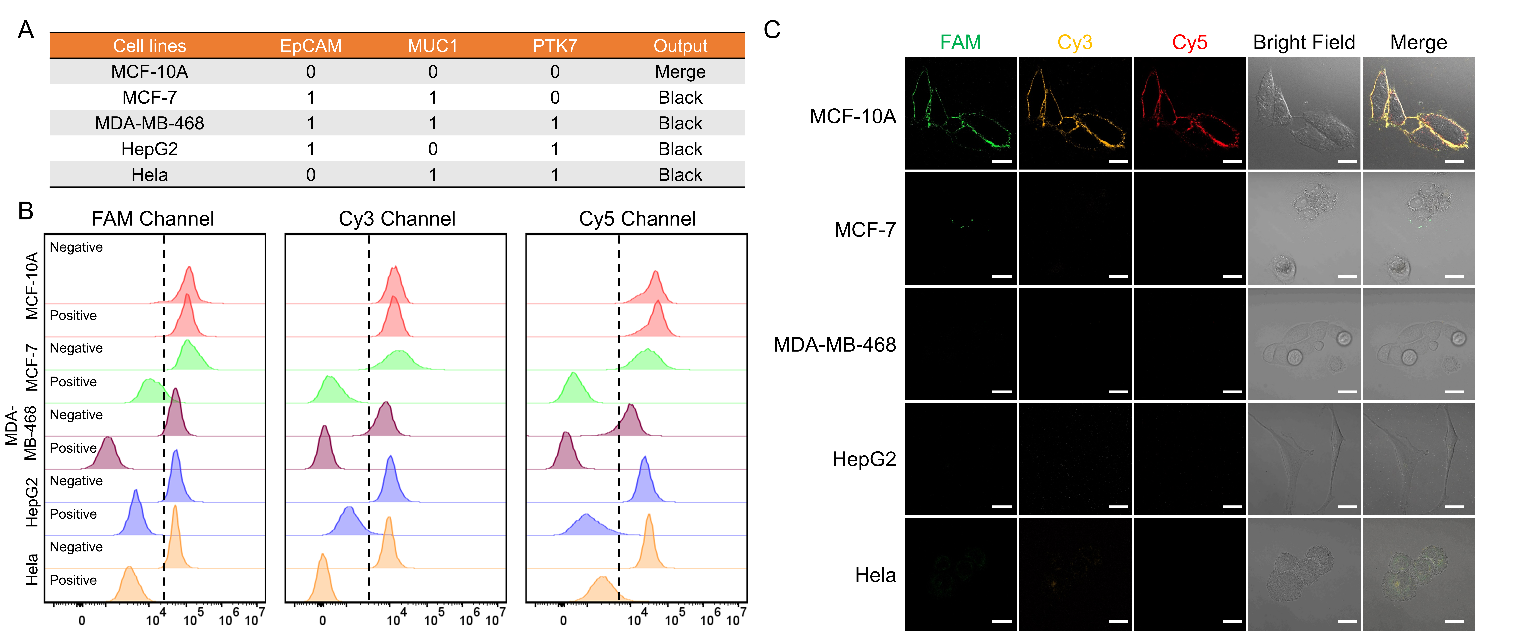


# Figure S27 The NOR-gate-based SDD-TRI system for cancer cell analysis. (A) The theoretical output values corresponding to different cell lines based on the NOR logic gate. Only the normal cell lines (MCF-10A) exhibited original state, while all other cancer cells showed the complete disassembly of CTDs. (B) Flow cytometry analysis for five different cells. (C) Confocal images of five different cell lines treated with NOR gate-based SDD-DRI system. Scale bar: 10 μm.

# Table S1 DNA sequences utilized for the construction of DNA scaffolds.

| Name | Sequence (5’-3’) | Modifications |
| --- | --- | --- |
| A1 | CCAACTTGCACTCACCATTCGCCAACTTGCACTCACCATTCG |  |
| A2 | CAGCTCGCTTCGATCTGTGGATGTAGC | 3’-Cy5 |
| A3 | GCTACATCCTGAGTGC |  |
| A4 | CCTATGGACATGACACGACGCGAATGGACAGATCGAAGCGAGCTG |  |
| A3’ | AGCTGGCTACATCCTGAGTGC |  |
| A4’ | CCTATGGACATGACACGACGCGAATGGACAGATCGAAGCG |  |
| B1 | GGAGTCACCATAGGTAGGAGCGGAGTCACCATAGGTAGGAGC |  |
| B2 | CAGCTCGTCGTGTCATGTGGTCTTAGC | 3’-Cy3 |
| B3 | GCTAAGACCTGACTCC |  |
| B4 | CGAATGGACAGATCGAAGCGCCTATGGACATGACACGACGAGCTG |  |
| B3’ | AGCTGGCTAAGACCTGACTCC |  |
| B4’ | CGAATGGACAGATCGAAGCGCCTATGGACATGACACGACG |  |
| C1 | TACCGTACATGACACCAGCAGTACCGTACATGACACCAGCAG |  |
| C2 | CAGCTCGTATCGGTACGTGGATCGAGC | 3’-FAM |
| C3’ | AGCTGGCTCGATCCTGTCATG |  |
| C4’ | GTCTAGCATTACGGTTAGCACTGCTGGACGTACCGATACG |  |

# Table S2 DNA sequences used for the molecular circuit and logic gates.

| Name | Sequence (5’-3’) | Modifications |
| --- | --- | --- |
| Input A | AGCTGGCTACATCCTGAGTGCAAGTTGGCGAATGGACAGATCGAAGCG |  |
| Input A’ | GCTACATCCTGAGTGCAAGTTGGCGAATGGACAGATC  GAAGCGAGCTG |  |
| Input B | AGCTGGCTAAGACCTGACTCCGCTCCTACCTATGGAC  ATGACACGACG |  |
| Input B’ | GCTAAGACCTGACTCCGCTCCTACCTATGGACATGAC  ACGACGAGCTG |  |
| Input C | AGCTGGCTCGATCCTGTCATGTACGGTACTGCTGGAC  GTACCGATACG |  |
| Input C’ | GCTCGATCCTGTCATGTACGGTACTGCTGGACGTACC  GATACGAGCTG |  |
| A4* | CGCTTCGATCTGTCCATTCGCGTCGTGTCATGTCCATAGG |  |
| B4* | CGTCGTGTCATGTCCATAGGCGCTTCGATCTGTCCATTCG |  |
| C4* | CGTATCGGTACGTCCAGCAGCAGATCGTAATGCCAATCGT |  |
| Input A* | CGCTTCGATCTGTCCATTCGCCAACTTGCACTCAGGATGATGCCAGCT | 5’-Cy5 |
| Variant 1 A3 | GCTACATCCT |  |
| Variant 1 A4 | GACAGATCGAAGCGAGCTG | 3’-BHQ2 |
| Variant 2 A3 | GCTACATCCTG |  |
| Variant 2 A4 | GGACAGATCGAAGCGAGCTG | 3’-BHQ2 |
| Variant 3 A3 | GCTACATCCTGA |  |
| Variant 3 A4 | TGGACAGATCGAAGCGAGCTG | 3’-BHQ2 |
| Variant 4 A3 | GCTACATCCTGAG |  |
| Variant 4 A4 | ATGGACAGATCGAAGCGAGCTG | 3’-BHQ2 |
| Variant 5 A3 | GCTACATCCTGAGT |  |
| Variant 5 A4 | AATGGACAGATCGAAGCGAGCTG | 3’-BHQ2 |
| Variant 6 A3 | GCTACATCCTGAGTG |  |
| Variant 6 A4 | GAATGGACAGATCGAAGCGAGCTG | 3’-BHQ2 |
| Variant 7 A3 | GCTACATCCTGAGTGC |  |
| Variant 7 A4 | CGAATGGACAGATCGAAGCGAGCTG | 3’-BHQ2 |
| Variant 8 A3 | GCTACATCCTGAGTGCA |  |
| Variant 8 A4 | GCGAATGGACAGATCGAAGCGAGCTG | 3’-BHQ2 |
| Variant 9 A3 | GCTACATCCTGAGTGCAA |  |
| Variant 9 A4 | GGCGAATGGACAGATCGAAGCGAGCTG | 3’-BHQ2 |
| Variant 10 A3 | GCTACATCCTGAGTGCAAG |  |
| Variant 10 A4 | TGGCGAATGGACAGATCGAAGCGAGCTG | 3’-BHQ2 |

# Table S3 DNA sequences used for cell imaging.

| Name | Sequence (5’-3’) | Modifications |
| --- | --- | --- |
| Sgc8c-probe | ATCTAACTGCTGCGCCGCCGGGAAAATACTGTACGGTTAGATTTTTTTTTTTTTTTTTTT | 3’-Cy5 |
| Sgc8c-S-trigger | ATCTAACTGCTGCGCCGCCGGGAAAATACTGTACGGTTAGATTTTTTTTTTTTTTTAGTTGGCGAATGGACAGATCGAAGCG |  |
| Sgc8c-H1 | CGCTTCGATCTGTCCATTCGCCAACTGACTTCCATAAGTTGGCGAATGGACA |  |
| Sgc8c-H2 | AGTTGGCGAATGGACAGATCGAAGCGTGTCCATTCGCCAACTTATGGAAGTC | 3’-Cy5 |
| Sgc8c-S-Input A (5 nt Spacer) | ATCTAACTGCTGCGCCGCCGGGAAAATACTGTACGGTTAGATTTTTAGCTGGCTACATCCTGAGTGCAAGTTGGCGAATGGACAGATCGAAGCG |  |
| Sgc8c-S-Input A (10 nt Spacer) | ATCTAACTGCTGCGCCGCCGGGAAAATACTGTACGGTTAGATTTTTTTTTTAGCTGGCTACATCCTGAGTGCAAGTTGGCGAATGGACAGATCGAAGCG |  |
| Sgc8c-S-Input A (15 nt Spacer) | ATCTAACTGCTGCGCCGCCGGGAAAATACTGTACGGTTAGATTTTTTTTTTTTTTTAGCTGGCTACATCCTGAGTGCAAGTTGGCGAATGGACAGATCGAAGCG |  |
| Sgc8c-S-Input A (20 nt Spacer) | ATCTAACTGCTGCGCCGCCGGGAAAATACTGTACGGTTAGATTTTTTTTTTTTTTTTTTTTAGCTGGCTACATCCTGAGTGCAAGTTGGCGAATGGACAGATCGAAGCG |  |
| Sgc8c-S-Random | ATCTAACTGCTGCGCCGCCGGGAAAATACTGTACGGTTAGATTTTTTTTTTTTTTTTTTTTCGTTACGCTATCGATGCGGAACTAAGCTACGCCAGTTAGTAACCTAGC |  |
| TCO1-probe | ACCAAACACAGATGCAACCTGACTTCTAACGTCATTTGGTGTTTTTTTTTTTTTTT | 3’-Cy3 |
| TCO1-S-trigger | ACCAAACACAGATGCAACCTGACTTCTAACGTCATTTGGTGTTTTTTTTTTTTTTTAGCTGGCTAAGACCTGACTCCGCTCC |  |
| TCO1-H1 | GGAGCGGAGTCAGGTCTTAGCCAGCTCTTAGAGATAAGCTGGCTAAGACCTG |  |
| TCO1-H2 | AGCTGGCTAAGACCTGACTCCGCTCCCAGGTCTTAGCCAGCTTATCTCTAAG | 3’-Cy3 |
| TCO1-S-Input B | ACCAAACACAGATGCAACCTGACTTCTAACGTCATTTGGTGTTTTTTTTTTTTTTTAGCTGGCTAAGACCTGACTCCGCTCCTACCTATGGACATGACACGACG |  |
| TCO1-S-Random | ACCAAACACAGATGCAACCTGACTTCTAACGTCATTTGGTGTTTTTTTTTTTTTTTTACGGACTTTACGGACGAAGCTGCTATCGACGCATGCTGCTGGCTATTG |  |
| EpCAM-S-Input A’ | CACTACAGAGGTTGCGTCTGTCCCACGTTGTCATGGGGGGTTGGCCTGTTTTTTTTTTTTTTTGCTACATCCTGAGTGCAAGTTGGCGAATGGACAGATCGAAGCGAGCTG |  |
| EpCAM-S-Random | CACTACAGAGGTTGCGTCTGTCCCACGTTGTCATGGGGGGTTGGCCTGTTTTTTTTTTTTTTTACGATGGGATAATTTCGTAGCGATCGGCAGGTCTAGGTACCGCGAATT |  |
| MUC1-S-Input B’ | GCAGTTGATCCTTTGGATACCCTGGTTTTTTTTTTTTTTTGCTAAGACCTGACTCCGCTCCTACCTATGGACATGACACGACGAGCTG |  |
| MUC1-S-Random | GCAGTTGATCCTTTGGATACCCTGGTTTTTTTTTTTTTTTGTCCCTATGGATGCAATCGTAGCTACAAGCCCTTACCGATACGTAATC |  |
| PTK7-S-Input C’ | ATCTAACTGCTGCGCCGCCGGGAAAATACTGTACGGTTAGATTTTTTTTTTTTTTTGCTCGATCCTGTCATGTACGGTACTGCTGGACGTACCGATACGAGCTG |  |
| PTK7-S-Random | ATCTAACTGCTGCGCCGCCGGGAAAATACTGTACGGTTAGATTTTTTTTTTTTTTTACGTCGGCTAGTCATACGTCAGCATAAACCGTCATCGGACGATATGGC |  |
